# Defects, (Sb, Er) Dopant Interactions, and Band Gap Engineering in Cs_2_AgInCl_6_ Double Perovskites

**DOI:** 10.1021/acs.jpcc.6c03164

**Published:** 2026-09-28

**Authors:** Inna A. Ivashchenko, Wojciech Piotrowski, Marcin Nyk, Lubomir D. Gulay, Volodymyr O. Yukhymchuk, Galina Gurieva, Andreas Eich, Susan Schorr, Beata Szreniawa, Oleg Yu. Khyzhun, Natalya Kurgan, Malgorzata Makowska-Janusik, Karina V. Lamonova, Katarzyna Matras-Postołek

**Affiliations:** † 272584 Cracow University of Technology, Faculty of Chemical Engineering and Technology, Warszawska St. 24, Cracow 31-155, Poland; ‡ Institute of Advanced Materials, 49567 Wroclaw University of Science and Technology, Wyb. Wyspiańskiego 27, Wrocław 50-370, Poland; § 187498 Lesya Ukrainka Volyn National University, Voli Ave. 13, Lutsk 43000, Ukraine; ∥ V.E. Lashkaryov Institute of Semiconductor Physics, National Academy of Sciences, Prospekt Nauki 41, Kiev 03028, Ukraine; ⊥ Helmholtz-Zentrum Berlin für Materialien Und Energie, Hahn-Meitner-Platz 1, Berlin 14-109, Germany; # Institut of Geological Sciences, Freie Universität Berlin, Kaiserswerther Str. 16-18, Berlin 14195, Germany; ∇ Frantsevich Institute for Materials Science, 226323 National Academy of Sciences of Ukraine, O. Pritsaka (Krzhizhanovsky) Str. 3, Kyiv 03-142, Ukraine; ○ G.V. Kurdyumov Institute of Metal Physics, 111230 National Academy of Sciences of Ukraine, Vernadsky St. 36, Kyiv 03-142, Ukraine; ◆ Faculty of Science and Technology, 69695 Jan Dlugosz University, Al. Armii Krajowej 13/15, Częstochowa 42-200, Poland; ¶ O. O. Galkin Donetsk Institute for Physics and Engineering, 28410 National Academy of Sciences of Ukraine, Nauky Ave. 46, Kyiv 03-028, Ukraine

## Abstract

In this work, the double perovskite Cs_2_AgInCl_6_ and its Sb^3+^- and Er^3+^-doped powders have been studied to understand how defects and dopants influence optical characteristics. Incomplete AgCl incorporation creates V_k_ and F centers, as well as defect-related In^+^ and Ag^–^ states, leading to photoluminescence (PL) bands at approximately 440, 610, and 630 nm, with the dominant peak at 630 nm. Cs_2_AgInCl_6_ exhibits a direct band gap of 4.29 eV, which is reduced by chlorine vacancies. Sb^3+^ doping results in weak PL, whereas Er^3+^ introduces visible-near-infrared (Vis-NIR) emission (525–1520 nm). Density functional theory (DFT) supports these observations, predicting a direct 4.42 eV gap for Cs_2_AgInCl_6_, narrowed by chlorine cation defects (3.3–3.9 eV), Sb^3+^ (3.41 eV), and Er^3+^ (3.97 eV) incorporation. Overall, the findings suggest that emission efficiency is limited by complex defect-dopant interactions; however, band gap tuning through doping indicates potential applications for wide-range photodetection.

## Graphical abstract

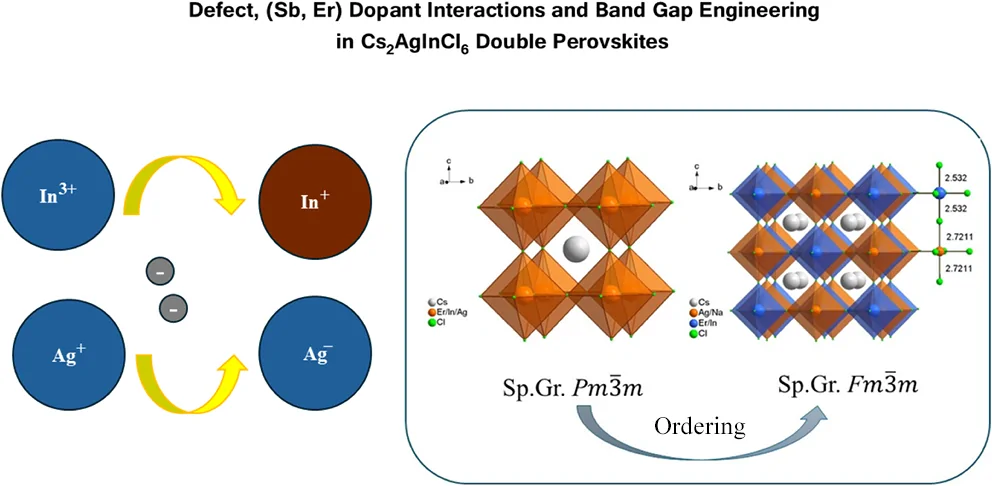

## 1 Introduction

Lead-free halide double perovskites (HDPs) have attracted considerable attention as environmentally friendly alternatives to lead halide perovskites because of their excellent chemical stability and promising optical properties. Among them, Cs_2_AgInCl_6_ is a particularly interesting material owing to its direct wide band gap, high structural stability, and potential applications in light-emitting devices, photodetectors, and photovoltaics. Rare-earth (RE) doping of Cs_2_AgInCl_6_ has been widely explored to extend its optical response into the NIR region and improve its functionality in optoelectronic applications.^1–12^

The optical properties of Cs_2_AgInCl_6_ remain under debate. While its luminescence is commonly attributed to self-trapped excitons (STEs) associated with Jahn–Teller distortion of the [AgCl_6_] octahedra, reported emission characteristics vary significantly in the literature, with PL maxima observed between approximately 395 and 607 nm under different excitation conditions.^5,9,13–18^ These discrepancies suggest that, in addition to STEs, defect-related states may contribute to the emission mechanism.

Defects are known to play an important role in halide materials by creating localized electronic states that affect charge trapping and radiative recombination. Recent studies have demonstrated the formation of V_k_ centers (halogen dimer hole traps), F centers, and defect-related trapping states in halide double perovskites, while XPS investigations have revealed charge redistribution around Ag, In, and halogen atoms associated with defect formation.^18–30^ Such defects may influence the optical response of Cs_2_AgInCl_6_ and contribute to the broad range of emission properties reported for this material.

In addition to defect engineering, cation doping provides an effective strategy for tailoring the luminescence of Cs_2_AgInCl_6_. Sb^3+^ ions can introduce ns^2–^-related electronic excitations and improve photostability, whereas Er^3+^ ions provide characteristic visible (Vis) and NIR emissions arising from *f*-*f* transitions.^19,20^ Therefore, understanding the interplay between intrinsic defects, Sb^3+^/Er^3+^ dopants, and the host lattice is essential for the further development of Cs_2_AgInCl_6_-based luminescent materials.

In this work, we investigate the structural, optical, and electronic properties of undoped, Sb^3+^-doped, and Er^3+^-doped Cs_2_AgInCl_6_. By combining XRD, Raman spectroscopy, XPS, photoluminescence measurements, and density functional theory (DFT) calculations, we examine the role of defects and dopant-induced states in luminescence and band gap modification, providing further insight into the emission mechanism of this double perovskite system.

## 2 Experimental Section

The microcrystalline powders for the investigation were obtained via precipitation and hydrothermal techniques. Details of the synthesis are described in the Supporting Information (SI) and the obtained samples are presented in Figures S2 and S3. In Table S1, it can be seen that the precipitate yield increases twofold after Sb^3+^ incorporation (from 32% for Cs_2_AgInCl_6_ to 66% for Sb-doped Cs_2_AgInCl_6_). When the Er^3+^ dopant level increases from 0.1 to 0.5 mol of ErCl_3_, the precipitate yield decreases from 60% to 29%. This indicates that Sb^3+^ incorporation improves the precipitate yield. Initially, Er^3+^ incorporation increases the practical yield when ErCl_3_ is 0.1 mol (60%), but when the chloride content is increased further, the practical yield decreases (29% for Cs_2_AgIn_0.5_Cl_6_·0.5ErCl_3_) and becomes lower than that for Cs_2_AgInCl_6_. Such a small practical yield could be explained by the limited solubility of AgCl in 36% HCl and its subsequent incorporation into the HDP crystal structure, as discussed in Section 3.1. For this reason, the hydrothermal technique was used, and, in that case, larger crystals were obtained. The obtained samples were investigated with X-ray powder diffraction (XRD), Raman spectroscopy, X-ray photoelectron spectroscopy (XPS), and UV–vis spectrophotometry, while single-crystal investigations were performed using single-crystal XRD (see the SI). Detailed information on sample synthesis and investigation methods is provided in SI.

## 3 Results and Discussion

### 3.1 XRD Results and Crystal Structure Analysis

The crystal structure of Cs_2_AgInCl_6_ has been refined as an ordered crystal structure with space group (Sp.Gr.) *Fm*3̅*m* ^31^ using the powder method (Figure 1). Rietveld refinements were performed to determine lattice parameters, phase purity, and to calculate structural parameters after doping of the samples. Sb^3+^ incorporation does not change the face-centered cubic structure but increases the unit-cell parameter compared to undoped Cs_2_AgInCl_6_ (Table 1). This agrees with the ionic radius of Sb^3+^ (0.86 Å) for octahedral coordination compared to 0.80 Å for In^3+^.^32,33^ A single-crystal approach for Cs_2_AgInCl_6_ yields two models: disordered (Sp. Gr. *Pm*3̅*m*) with *a* = 5.23373(7) Å (*R*1 = 0.0279) and ordered (Sp.Gr. *Fm*3̅*m*), with a unit cell parameter twice as large, *a* = 10.4695(2) Å, and a slightly lower *R*1 value of 0.0178 (see Tables 2–3 and Tables S2–S5). Both structures have group-subgroup relations, as previously established in refs^34^ and^35^ (Figure 1). Sb incorporation does not create a strong Jahn–Teller distortion; however, Sb increases the yield of the synthesis, the stability of the powder, and the symmetry of the unit cell, making the ordered structure preferred (Figure S4, Table 1). This also influences the optical properties of the Sb-doped Cs_2_AgInCl_6_ powder samples (see Section 3.5 and 3.6).

**Figure 1:**
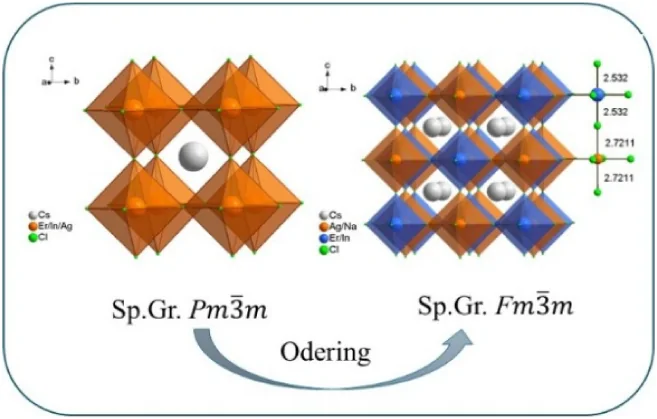
Models of the crystal structures for Cs_2_AgInCl_6_.

**Table 1:** Composition of Synthesized Samples by Precipitation Technique and Their Crystal Parameters, Investigated by Powder (Powd.) or Single Crystal (S.Cr.) Methods

Compositions	Sp.Gr.; lattice parameters, Å	Method of calculation
Cs_2_AgInCl_6_	*Fm*3̅*m*; *a* = 10.4806(2)/10.4695(2) Å*Pm*3̅*m*; *a* = 5.23373(7)	powd.^b^/s.cr.^b^ s.cr.^b^
Cs_2_AgIn_0.9_Sb_0.1_Cl_6_	*Fm*3̅*m*; *a* = 10.5060(2)	powd.^a^
Cs_2_AgIn_0.9_Er_0.1_Cl_6_ (CsAg_0.5_In_0.45_Er_0.05_Cl_3_)	*Pm*P3̅*m*; *a* = 5.24204	powd.^a^
Cs_2_AgIn_0.7_Er_0.3_Cl_6_ (CsAg_0.5_In_0.35_Er_0.15_Cl_3_)	*Pm*P3̅*m*; *a* = 5.24234	powd.^a^
Cs_2_AgIn_0.5_Er_0.5_Cl_6_ (CsAg_0.5_In_0.25_Er_0.25_Cl_3_)	*Pm*3̅*m*; *a* = 5.24249	powd.^a^
Cs_2_AgIn_0.3_Er_0.7_Cl_6_	two-phase with AgCl	powd.

a The crystals were too small to be selected for crystal-structure investigation by the single-crystal method.

b Crystals for crystal-structure investigation were obtained using the hydrothermal technique.

**Table 2:** Feeding Concentration (in Parenthesis) and Analytical Compositions of the Obtained Powder Samples^a^

Composition	Cs (at.%)	Ag (at. %)	In (at. %)	Sb (at. %)	Er (at. %)	Cl (at.%)
Cs_2_AgInCl_6_	20.27 (20.0)	7.4 (10.0)	10.4 (10.0)	-	-	59.5 (60.0)
Cs_2_AgIn_0.9_Sb_0.1_Cl_6_	20.0 (20.0)	8.5 (10.0)	10.0 (9.0)	2.0 (1.0)	-	59.5 (60.0)
Cs_2_AgIn_0.9_Er_0.1_Cl_6_	21.5 (20.0)	8.8 (10.0)	10.1 (9.0)	-	0.6 (1.0)	59.0 (60.0)
Cs_2_AgIn_0.7_Er_0.3_Cl_6_	21.7 (20.0)	8.5 (10.0)	10.0 (7.0)	-	1.3 (3.0)	58.5 (60.0)
Cs_2_AgIn_0.5_Er_0.5_Cl_6_	20.4 (20.0)	8.1 (10.0)	10.0 (5.0)	-	2.5 (5.0)	59.0 (60.0)

a The EDAX errors were within 0.4%.

**Table 3:** Batch (in Parenthesis) and Analytical Compositions of the Obtained Single Crystals

Composition	Cs (at.%)	Ag (at.%)	In (at.%)	Cl (at.%)	O (at.%)
Cs_2_AgInCl_6_ (s. cr.)	19.0 (20.0)	9.1 (10.0)	9.3 (10.0)	62.6 (60.0)	-
Cs_2_InCl_5_·H_2_O (s. cr.)	14.0 (20.0)	-	8.6 (10.0)	48.8 (50.0)	19.7 (20.0)
Cs_2_InBr_5_·H_2_O (powder)	21.7 (20.0)	-	9.0 (10.0)	68.7 (60.0)	19.8 (20.0)

During the synthesis, we presume that Cs_2_InCl_5_·H_2_O could be obtained when there is a deficiency of AgCl at the first stage of HDP formation (Figure 2, Table 3):

$${\mathrm{2CsCl}+\mathrm{InCl}}_{3}+H_{2}{O=\mathrm{Cs}}_{2}{\mathrm{InCl}}_{5}{\mathrm{\cdot H}}_{2}O$$

with the subsequent formation of the HDPs (Table 3):

$${\mathrm{Cs}}_{2}{\mathrm{InCl}}_{5}+{\mathrm{AgCl}=\mathrm{Cs}}_{2}{\mathrm{AgInCl}}_{6}$$

**Figure 2:**
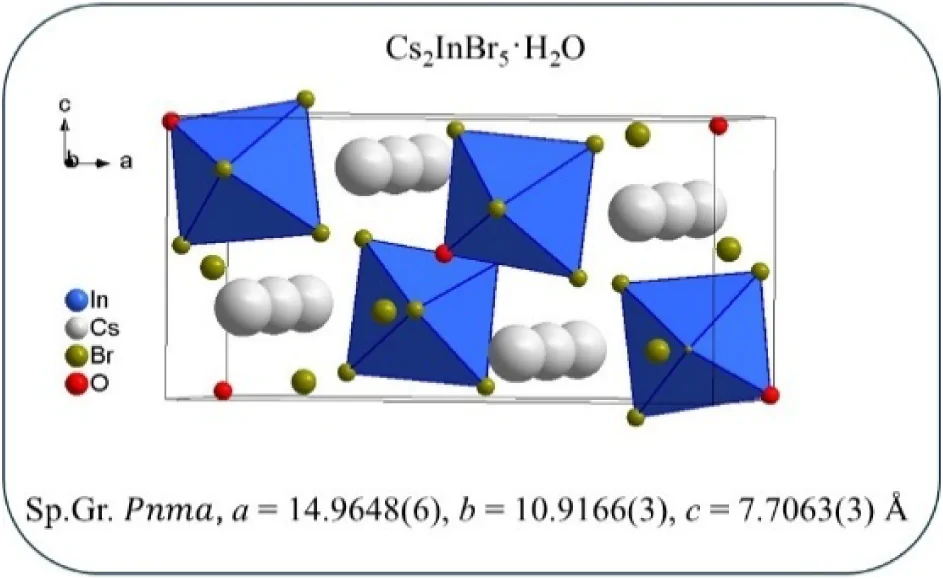
Models of the crystal structures for Cs_2_InBr_5_·H_2_O.

The XRD patterns of the Er-doped Cs_2_AgInCl_6_ samples were refined in the cubic structure (Sp.Gr. *Pm*3̅*m*) with a statistical distribution of the Ag^+^ (1.15 Å), In^3+^ (0.80 Å), and Er^3+^ (0.89 Å) cations (Tables S6–S8, Figure S5). The unit cell parameter increases from *a* = 5.24204(9) Å for Cs_2_AgIn_0.9_Er_0.1_Cl_6_ sample to *a* = 5.24249(5) Å for Cs_2_AgIn_0.5_Er_0.5_Cl_6_ sample, which agrees with the larger ionic radius of Er^3+^ (0.89 Å) compared with the smaller In^3+^ (0.80 Å). We conclude that a disordered structure is preferable for the incorporation of Er ions into the Cs_2_AgInCl_6_ structure. However, it should be noted that the samples with 0.7 mmol of Er dopant exhibited two phases due to an AgCl impurity (Figure 3, Table 1) and the 0.5 mmol ErCl_3_ dopant gave the lowest yield (Table S1). This indicates that Er^3+^ incorporation increases structural instability and proceeds smoothly up to 0.3 mmol of ErCl_3_. We presume that this concentration is optimal for Cs_2_AgInCl_6_ synthesis because of the difference in the radii of the cations participating in HDP structure formation, with the large Ag^+^ (1.15 Å, C.N. = 6). A high concentration of Er increases the probability of a statistical distribution of the cations in the structure of doped Cs_2_AgInCl_6_ with the following consequences for optical properties (see Section 3.5 and 3.6).

**Figure 3:**
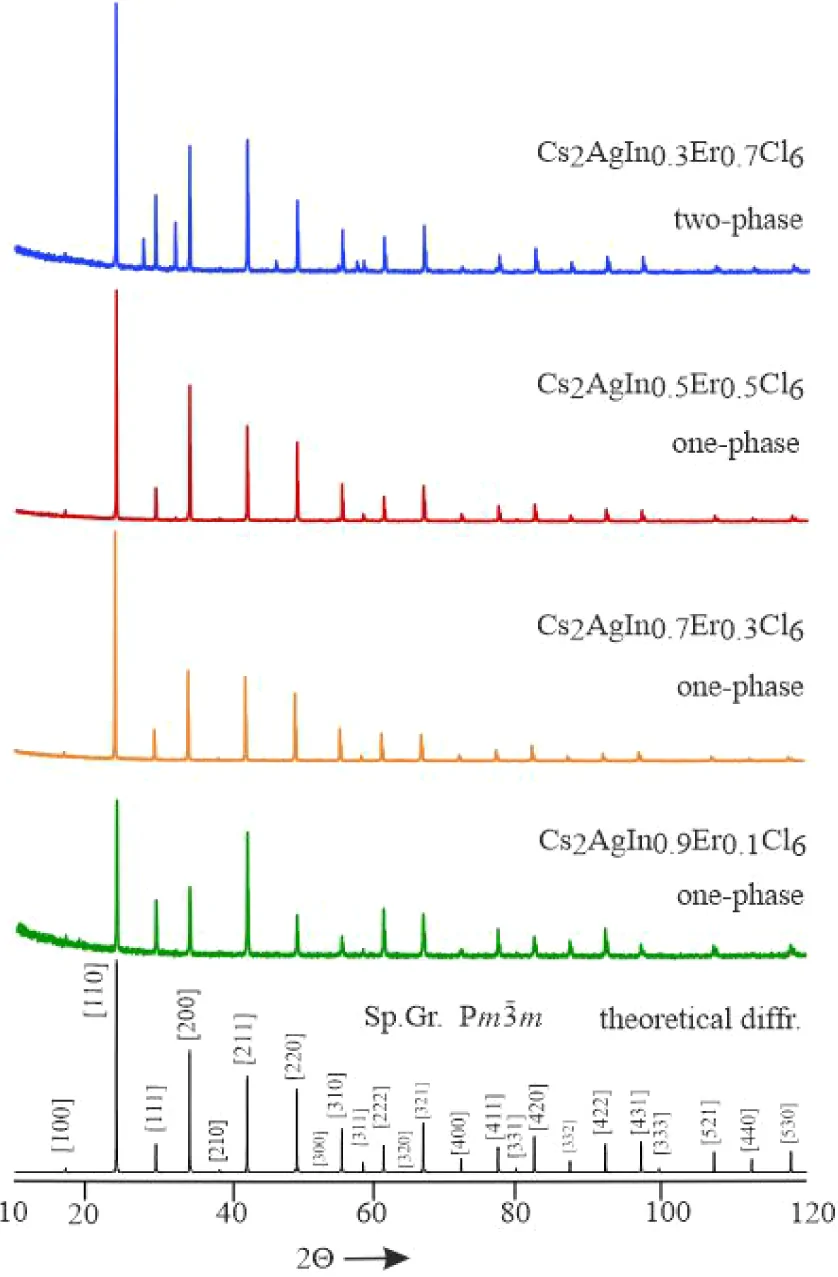
XRD results of the Er-doped Cs_2_AgInCl_6_ powder samples.

The EDS results (Tables 2, 3) show the lack of Ag^+^ and Cl^–^ due to the incomplete dissolution of AgCl during synthesis (traces can be seen in Figure S6). This can be explained by differences in the ionic radii of the cations and the difficulty of AgCl incorporation into the HDP structure (see DFT calculation results in Section 3.7). The In concentration is higher than the calculated value, and all samples contain less than the feeding amount of Er^3+^. Such low concentrations of RE dopants agree with ref.^20^ for Yb^3+^ and could be explained by the difficulty of RE incorporation. That is why the syntheses of the Er-doped Cs_2_AgInCl_6_ were performed for 5 h. The Cs_2_InCl_5_·H_2_O composition was established during crystal-structure determination using the single crystal method (Table 3, Figure S7b, Tables S9–S12). Figure S7a shows traces of unreacted AgCl on the surface of the Cs_2_AgInCl_6_ single crystal, which served as the basis for the investigation of the crystal structure of the HDP (Tables S2–S5) and was discussed earlier (see Section 3.1).

Accidentally, we obtained a similar bromine-containing compound, Cs_2_InBr_5_·H_2_O (Figure 2; Figure S7 c, d; Table 3) during Cs_2_AgInBr_6_ and Cs_2_NaInBr_6_ syntheses and calculated its crystal structure by the powder method (Tables S13–S15, Figure S8). We presume that the formation of Cs_2_InCl­(Br)_5_·H_2_O is energetically preferable (see DFT calculation Section 3.7) and that it is necessary to increase the temperature or pressure to incorporate Ag or Na halides into the structure to obtain HDPs. In the end, Cs_2_AgInBr_6_ and Cs_2_NaInBr_6_ were not obtained probably due to the difficulties of ABr (A = Na, Ag) incorporation into the Cs_2_InBr_5_ crystal structure. Their syntheses require further improvement.

### 3.2 Raman Spectroscopy

According to the selection rules for HDPs A_2_B^I^B^III^X_6_, the Raman spectra include four vibrational modes: A_1g_, E_g_, and 2F_2g_, which describe the full vibrational representation like Γ_R_ = A_1g_ ⊕ E_g_ ⊕ 2F_2g_.^36^ All these vibrational modes are manifested both in the Raman spectra of the undoped compounds, which we studied in detail in our previous work,^31^ and in the spectra of the Er^3+^, Sb^3+^) compounds, which were studied in this work and are shown in Figure 4.

**Figure 4:**
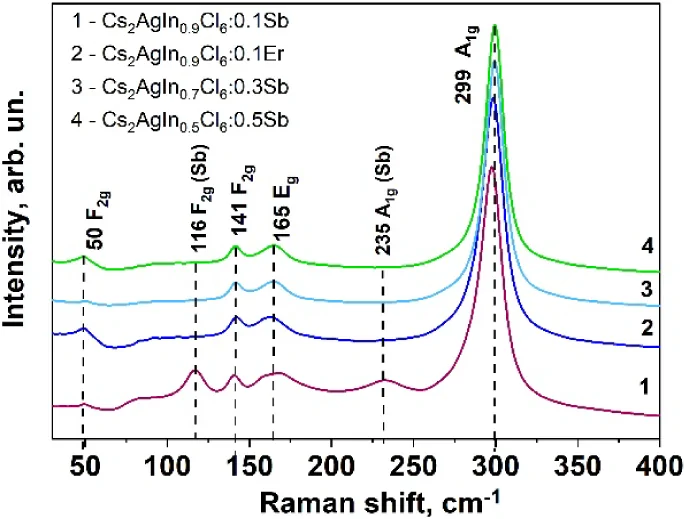
Raman spectra of Cs_2_AgInCl_6_ compounds doped with 1 at. % Sb^3+^ (curve 8) and with 1 at. %, 3 at. %, and 5 at. % Er^3+^ (curves 11, 10, and 9, respectively).

As can be seen in Figure 4, all four vibrational modes are present in the spectra: one symmetric stretching mode (A_1g_) at 299 cm^–1^, a doubly degenerate asymmetrical stretching mode (E_g_) at 165 cm^–1^, and two triply degenerate vibrational modes (F_2g_) at 50 cm^–1^ and 141 cm^–1^. The band at 50 cm^–1^ corresponds to the translational motion of Cs. The band at 141 cm^–1^ is a bending vibrational mode arising from the motion of two Cl atoms on either side of a fixed In or Ag atom, changing the corresponding Cl–In­(Ag)–Cl angle. Notably, the frequency of this bending mode does not change or may even increase with increasing temperature in the perovskites.^25,36^ In contrast, the other modes (A_1g_ and E_g_ bands) usually display a substantial decrease in frequency as the temperature rises, owing to bond elongation.

However, for the bending vibrational mode, the force constant may increase with increasing temperature, possibly due to alterations in the angles within the Cl–In­(Ag)–Cl system; this unusual behavior could help correctly identify this vibrational mode, as there are inconclusive reports in the literature regarding the interpretation of bands in the Raman spectra of Cs_2_AgInCl_6_, Cs_2_NaInCl_6_, and Cs_2_AgSbCl_6_.^37,38^

This is why we investigated how the band frequency positions change with temperature. A representative frequency dependence of the bands on temperature for 3 at. % Er-doped Cs_2_AgInCl_6_ (spectrum 10 in Figure 4) is shown in Figure 5. Other Raman spectra of 1 and 5 at. % Er-doped Cs_2_AgInCl_6_ are shown in Figure 4 (spectra 9 and 11), and they almost coincide with spectrum 10. With an increase in the Er concentration from 1 to 5 at. %, only A_1g_ and E_g_ bands shift slightly (∼1 cm^–1^) to the low-frequency side. Such a shift may be caused by the incorporation of Er^3+^ in place of In^3+^ and by a decreased effective force constant in these vibrational modes. At the same time, the bands F_2g_ do not change, which indicates greater rigidity of the bonds in the bending vibrational mode.

**Figure 5:**
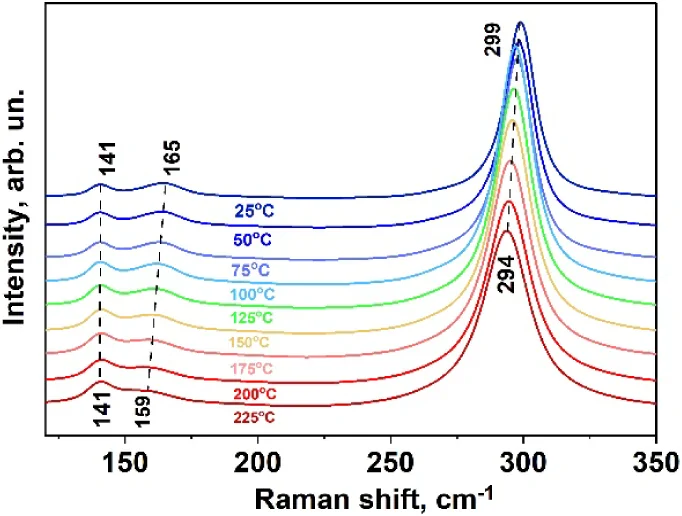
Raman spectra of sample 10, recorded as its temperature varied from 25 to 225 °C.

Spectrum 8 (Figure 4) corresponds to the Sb-doped Cs_2_AgInCl_6_ sample, and two additional bands at 235 and 116 cm^–1^ appear; the half-widths of the A_1g_ and E_g_ bands increase with a simultaneous low-frequency shift. The appearance of two new bands may indicate vibrations of the [SbCl_6_]^3–^ octahedra due to In^3+^ substitution on Sb^3+^. Since Sb and In differ in their properties (mass, electronic structure, bond stiffness), the vibrational modes of [SbCl_6_]^3–^ have significantly lower frequencies.

### 3.3 XPS Investigation

In the present XPS measurements, we strictly use the same apparatus and techniques as those we employed earlier for studies of the related perovskite chloride CsPbCl_3_.^39^ The XPS spectra were excited with an Al Kα X-ray source (see the SI for more details). The XPS survey spectrum data for the double perovskite Cs_2_AgIn_0.9_Sb_0.1_Cl_6_ are presented in Figure 6. It is evident that all features characteristic of XPS are well ascribed to the chemical elements composing the sample. The exceptions are the XPS O 1*s* and C 1*s* core levels associated with oxygen-bearing species and hydrocarbons adsorbed on the sample surface because of its contact with laboratory air prior to the XPS measurements. However, the relative intensities of the XPS O 1s and C 1s core-level spectra are rather low, considering that Cs_2_AgIn_0.9_Sb_0.1_Cl_6_ under study was in powder form.

**Figure 6:**
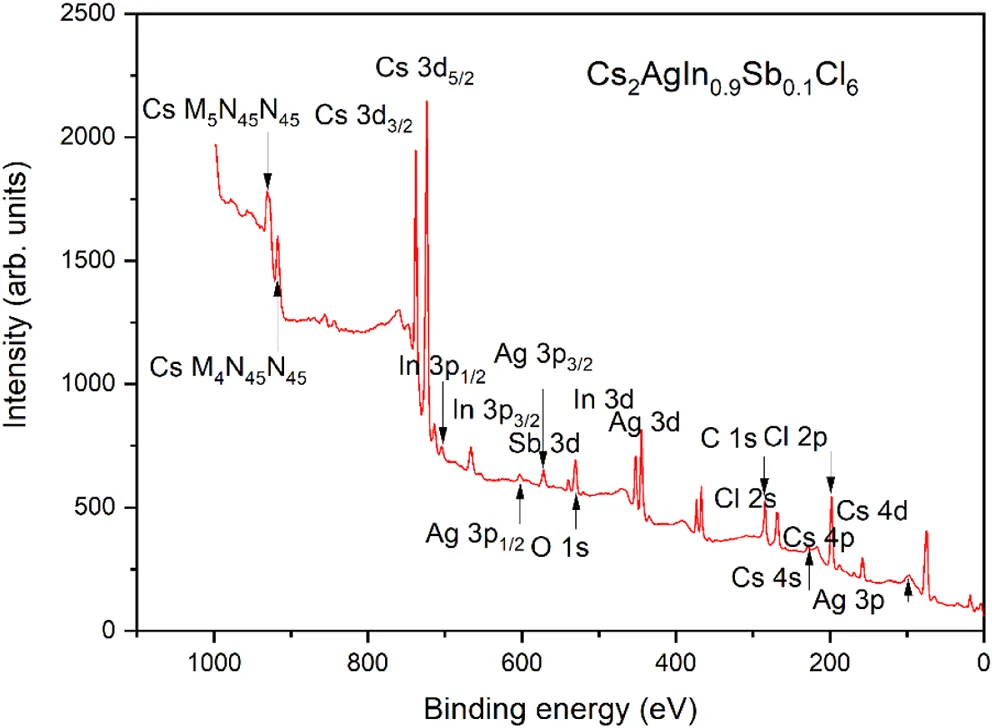
Survey XPS spectrum of Cs_2_AgIn_0.9_Sb_0.1_Cl_6_. Detailed XPS core-level spectra for Cs 3*d*, In 3*d*, Sb 3*d*, Ag 3*d*, and Cl 2*p* are presented in Figure S9.

The most informative and detailed XPS core-level spectra of the elements comprising the Cs_2_AgIn_0.9_Sb_0.1_Cl_6_ sample are presented in Figure S9, while the values of their binding energies are listed in Table S16. The data indicate that we have detected charge transfer around the In^3+^ ions. The binding energy of the In 3d_5/2_ core-level electrons in this sample is about 445.30 ± 0.08 eV, which is much bigger than in metallic In (ca. 443.8 eV)^40^ and is close to that in In_2_O_3_ (ca. 445.0 eV).^40^ However, it is much smaller than that in InCl_3_ (ca. 446.45 eV).^40^ The binding energy of the Ag 3d_5/2_ core-level electrons in Cs_2_AgIn_0.9_Sb_0.1_Cl_6_ is 367.89 ± 0.08 eV, which is slightly smaller than in metallic Ag (ca. 368.2 eV^41^). Therefore, we have detected a small charge transfer toward Ag atoms in the Cs_2_AgIn_0.9_Sb_0.1_Cl_6_ sample. Further, the binding energy of the Cs 3d_5/2_ core-level electrons (about 724.08 ± 0.08 eV, Table S16) in the studied sample indicates that Cs atoms also accept electrons, since in pure Cs this binding energy value is about 726.0 eV.^41^ On the other hand, the binding energy of Cl 2*p* electrons is 198.7 eV, close to that in NiCl_2_.^41^ The XPS valence-band spectrum (Figure S9) reveals the existence of two broad bands: the upper one centered at about 4.9 eV is formed mainly by Cl 3*p* electronic states, as revealed by the presented DFT calculations (see Section 3.7).

Defects influence the valence states of Ag^+^ (changing on Ag^0^, Ag^–^), In^3+^ (In^+^ formation), and Cl_2_^–^ dimers. These valence states were confirmed by our XPS results and agree with refs^28^ and^29^ It should be noted that, while the XPS results indicate charge redistribution around the Ag and In ions, they do not provide direct proof of specific defect species (see the Conclusions section for more details).

### 3.4 Optical Absorption and Excitation Spectra

A broad excitation band observed at 275–340 nm (4.5–3.60 eV), centered at ∼295 nm (Eg ∼ 4.2 eV), was detected in the measured absorption spectra and can be considered the band-gap absorption of Cs_2_AgInCl_6_ (Figure 7a,d). This result aligns with refs 5–7 and our DFT calculations (see Section 3.7, DFT calculation results). Additionally, weaker absorption peaks are observed at ∼370 nm and ∼490 nm. Doping with Sb red-shifts the absorption spectrum to 360–394 nm (3.44–3.14 eV), and an additional weaker band exists at ∼298 nm (Figure 7b). For the Er-doped samples, an excitation band is at ∼310 nm (∼4.01 eV), and there is a very weak peak at ∼490 nm (Figure 7b). In all absorption spectra (Figure 7d), there is a band at 225 nm that coincides exactly with the C band of In^+^.^42–44^ Also, the absorption bands at ∼290 nm and ∼318 nm for Cs_2_AgInCl_6_ (Figure 7a) can be attributed to the A band of In^+^.^42–44^

**Figure 7:**
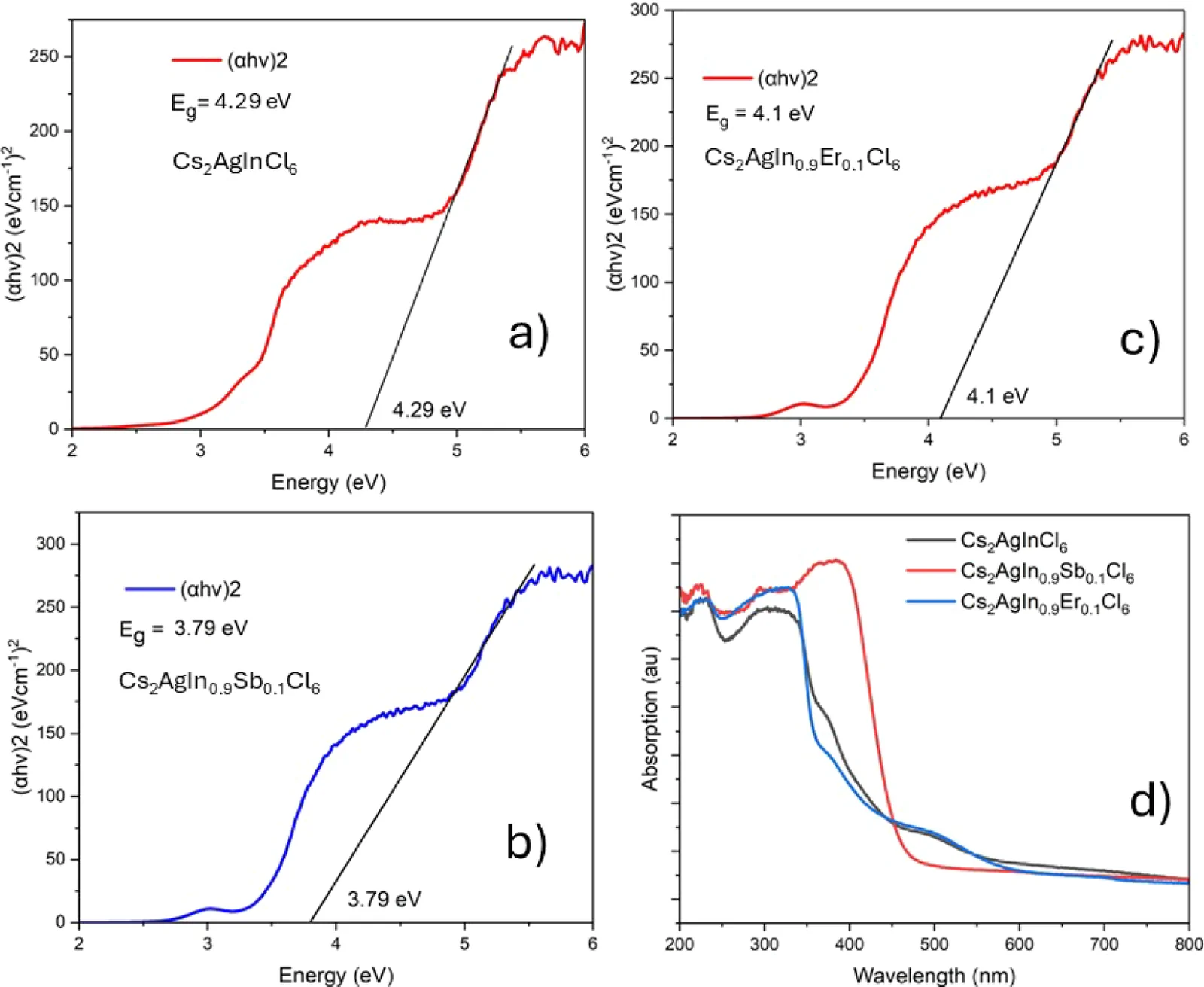
Tauc plots of the Cs_2_AgInCl_6_ (a), Sb-doped Cs_2_AgInCl_6_ (b), and 0.1 Er-doped Cs_2_AgInCl_6_ (c) samples with direct band gaps; d) absorption spectra of Cs_2_AgInCl_6_, Sb-doped Cs_2_AgInCl_6_, and 0.1Er-doped Cs_2_AgInCl_6_.

Measurement of the band gap was performed considering ref 45. The Tauc plots resemble those of semiconductors, showing notable absorption of sub-band gap photons. This is typical for defective or doped materials, which can introduce intra-band gap states that appear in the absorption spectrum as an additional, broad absorption band. The obtained E_g_ values align very well with the DFT calculations (see Section 3.7).

The measured excitation spectra (Figure 8a,b,c,d) for Cs_2_AgInCl_6_ and Sb- or Er-doped Cs_2_AgInCl_6_ samples agree with the absorption spectra. The excitation spectrum for Cs_2_AgInCl_6_ is broad, with three not-sharp maxima: the first at ∼278 nm and, equal to it, the second at ∼312 nm, which merge into a 300 nm band, and the third at ∼370 nm, which is the lowest-energy band.

**Figure 8:**
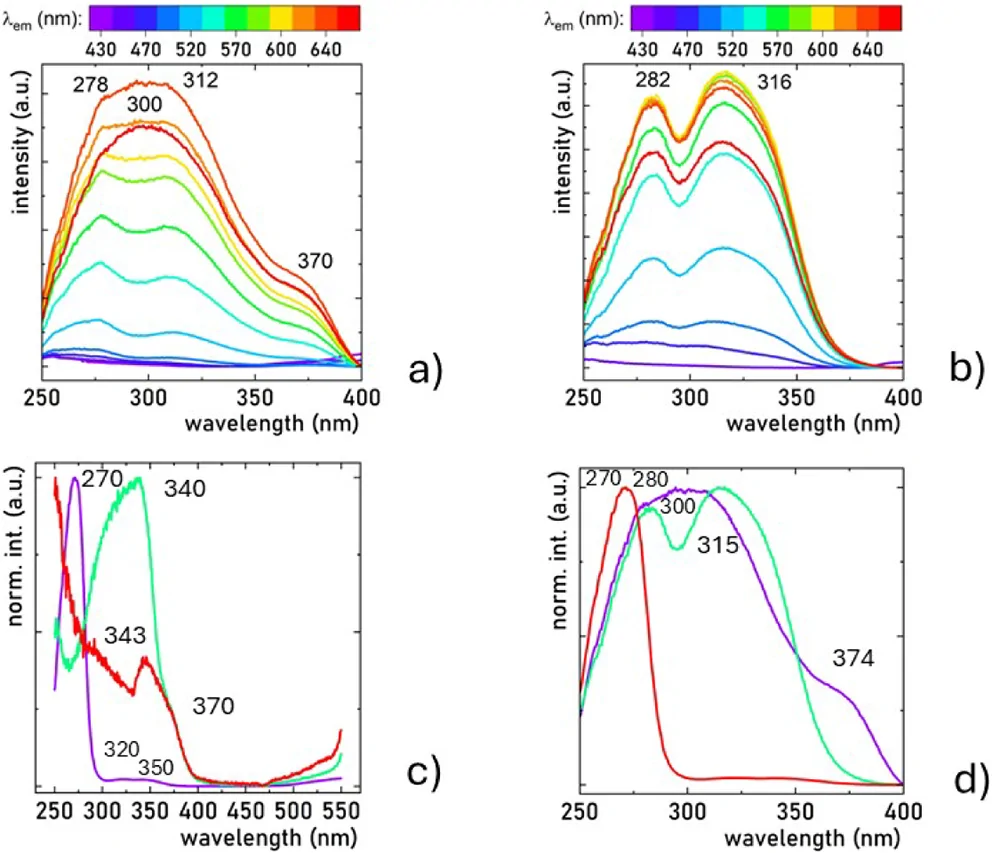
Influence of λ_em_ on the excitation spectra of (a) Cs_2_AgInCl_6_; (b) Cs_2_AgInCl_6_:0.1% Sb; (c) influence of Er^3+^ concentration on the excitation spectra of Cs_2_AgInCl_6_:*x*% Er^3+^ for λ_em_ = 630 nm; (d) comparison of the excitation spectra of undoped Cs_2_AgInCl_6_ with Er^3+^-doped and Sb^3+^-doped counterparts excited by λ_em_ = 630 nm.

Comparing these wavelengths with those presented in the literature for the Ag^–^ 5*s* ^2^ ion, we conclude that, according to refs 21–25 , the measured absorption bands correspond to the C-band at 276 nm, the B-band at ∼320 nm, and the A-band at ∼370 nm.

The Sb-doped excitation spectra exhibit more distinct intense bands at 260 nm (low intensity) and at 282 nm and ∼316 nm with equal intensities, which can be attributed to the C-band, B-band, and A-band, respectively.^46^ Maxima at 300 and 370 nm are absent compared to the undoped sample.

The excitation spectra of the Er-doped samples have completely different structures (Figure 8c,d): for the lowest Er concentration (1 at. % Er), the most intense band is at 270 nm, corresponding to Ag^–^ excitation in the sample, while very small bands at 320–350 nm correspond to excitation of Er ions and become dominant at higher Er concentrations (3 at. % and 5 at. %). This can be explained by the participation of Er^3+^ electrons in the formation of the conduction band of the sample (see DFT calculation results, Section 3.7), which is why the excitation spectra change significantly with increasing Er^3+^ concentration.

### 3.5 Photoluminescence Properties

We compared the PL of undoped Cs_2_AgInCl_6_ and Sb- or Er-doped Cs_2_AgInCl_6_ and observed differences between them (Figure 9a,b; Figure S10a,b,c). The Cs_2_AgInCl_6_ PL spectrum under 270 nm excitation has three maxima with different intensities: ∼440 nm (the lowest and very weak band), ∼610 nm (low intensity), and ∼640 nm (the highest intensity). The ∼440 nm band can be attributed to In^+^ ion presence, due to electron density transfer from Cl to In^3+^, induced by the lack of Ag^+^ in the samples. The In^+^ ion fulfills many different roles: it can substitute Ag^+^ in the crystal structure, it provokes Jahn–Teller distortions of the [Ag^+^(In^+^)­Cl_6_]^5–^ octahedra, and it forms PL bands at ∼ 450 and ∼620 nm; these results are in line with.^8^ The low-intensity PL at 610 nm agrees with literature data reporting 607 nm PL under 270 nm excitation in Cs_2_AgInCl_6_.^5,11,13^ Moreover, in^25^ the emission at 678 nm for KCl:Ag^–^ was obtained under 330, 283, and 278 nm excitation. The authors explain that absorption at these wavelengths arises from the same center (Ag^–^) and these suggestions are consistent with our results. In Figure S10a, the PL obtained for Cs_2_AgInCl_6_ under 270 and 340 nm excitation is mostly the same, with peaks at ∼610 and ∼640 nm (the highest peak), which agrees with the reddish-brown appearance of the sample under 254 nm light (see Figure S2).

**Figure 9:**
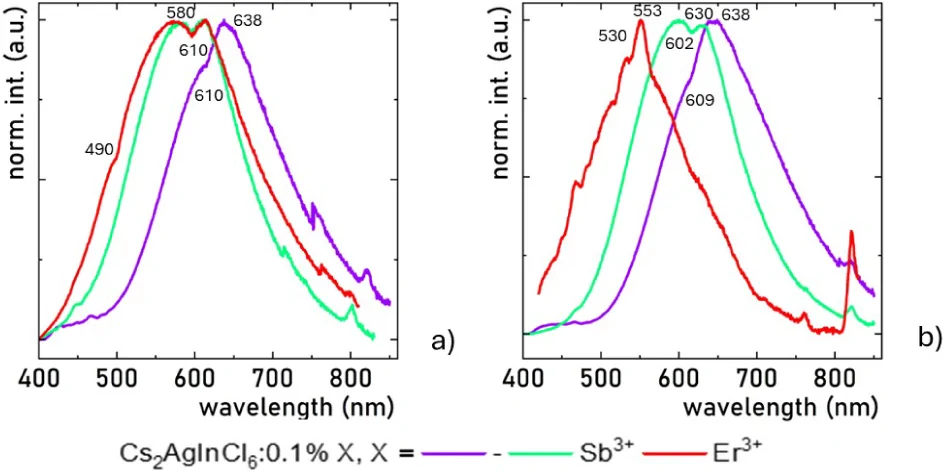
Comparison of the emission spectra of undoped Cs_2_AgInCl_6_ with Er^3+^-doped and Sb^3+^-doped counterparts excited by (a) λ_exc_ = 270 nm; (b) 340 nm. The violet curve corresponds to the undoped sample, the green curve to the 1 at. % Sb-doped sample, and the red curve to the 1 at. % Er-doped sample.

In the Er-doped samples under 340 nm radiation, there is energy transfer from Cs_2_AgInCl_6_ matrix to the Er^3+^ ions, which also emit radiation in the Vis- and NIR regions, namely, ^2^H_11/2_ → ^4^I_15/2_ (530 nm) and ^4^S_3/2_ → ^4^I_15/2_ (550 nm), because of energy transitions in the *f*-shells of Er^3+^ ions (Figure S10 b). This mechanism aligns with our earlier investigation of the HDPs doped with Er3+^31^ and other investigations of Er-doped samples.^47,48^ Under 270 nm excitation, the highest bands are at ∼595 nm and ∼630 nm, which agree with the PL of Cs_2_AgInCl_6_ and could be explained in the same way, by In^+^ and Ag^–^ (5*s* ^2^) excitation.^22–30^

For the Sb-doped samples (Figure S10c), we did not observe intense PL at 450 nm, as opposed to Cs_2_NaInCl_6_ ^46^ under 320 nm excitation. As the PL spectra for Sb-doped Cs_2_AgInCl_6_ are mostly similar under different excitations, with PL bands at 580 and 610 nm (λ_ex_ = 270 nm) and 600 and 630 nm (λ_ex_ = 340 nm) (Figure S10c), we conclude that there is no Sb emission of Sb^3+^, as it would otherwise be very intense. However, there is emission from In^+^ and Ag^–^ as 5*s* ^2^ ions, as in undoped Cs_2_AgInCl_6_. Nevertheless, as can be seen in Figure 9b, there is a red shift for undoped Cs_2_AgInCl_6_ compared to Sb-doped Cs_2_AgInCl_6_ and does not agree with the measured absorption spectra (Figure 7) (*E*_abs_ (Cs_2_AgInCl_6_) ∼ 4.29 eV; *E*_abs_ (Cs_2_AgIn_0.9_Cl_6_·0.1Sb) ∼ 3.79 eV). This discrepancy can be explained by the formation of hole-trapping centers (Cl_2_^–^ dimers), whereas In^3+^ and Ag^+^ play the role of electron-trapping centers.^22–30^ These factors decrease the band gap and are supported by DFT calculation results (see Section 3.7).

To avoid competition between two PL excitation channels, such as Cs_2_AgInCl_6_ and Er^3+^ ions, the samples were irradiated at an 808 nm excitation wavelength, which corresponds to transitions from the ^4^I_15/2_ level to the ^4^I_9/2_ level within Er^3+^. The Stokes luminescence, with a peak at 1540 nm (Figure 10), corresponds to the ^4^I_13/2_ → ^4^I_15/2_ transition in Er^3+^ ions. It should be noted that the band intensity increases in samples with 1 at. % Er and 3 at. % Er, and then decreases in the sample with 5 at. % Er. This is caused by concentration quenching, which results from an increased probability of energy transfer between Er^3+^ ions and nonradiative transitions. A similar trend was observed in our previous works.^31,47,48^

**Figure 10:**
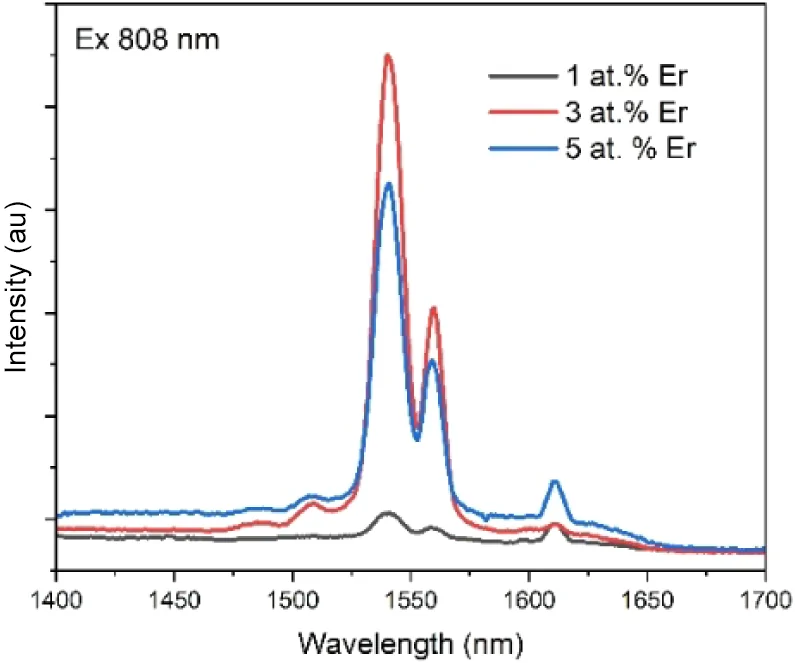
Emission spectra of the Er-doped Cs_2_AgInCl_6_ samples at λ_exc_ = 808 nm, with a peak at 1540 nm, corresponding to the ^4^I_13/2_ → ^4^I_15/2_ transition in the Er^3+^ ions. Synthesized samples based on Cs_2_AgInCl_6_ HDPs demonstrated only Stokes (down-conversion) luminescence.

### 3.6 Factors Influencing the PL Properties of Cs_2_AgInCl_6_ and Doped Samples

The composition of the HDP, undoped Cs_2_AgInCl_6_ and Sb- or Er-doped Cs_2_AgInCl_6_, the synthesis method (precipitation or hydrothermal), and the synthesis duration (2–5 h) determine the surface quality of the microcrystals, the phase composition of the samples, and cation and anion defects. A lack of Cl ions stimulates the formation of hole-trapping centers (Cl_2_^–^ dimers). A deficiency of Ag^+^ ions in the sample promotes their substitution by In^+^ ions, which form because the In^3+^ ions act as electron-trapping centers and form In^+^ ions. The mechanism of electron trapping explains the possibility of Ag^–^ formation from Ag^+^ and Ag^0^. In^+^ and Ag^–^ ions have a 5*s* ^2^ electron configuration; the ground state is ^1^S_0_, and the excited states are ^3^P_0_, ^3^P_1_, ^3^P_2_, and ^1^P_1_, corresponding to the 5*s* ^1^ *p* ^1^ electron configuration because of the Jahn–Teller distortion of the octahedra in the HDP crystal structure (Figure 11). The ^1^S_0_ ↔ ^1^P_1_, ^3^P_1,2_ transitions (C, B, A bands) become partially permitted and can be observed in optical spectra. It should be underlined that we describe the Ag-related emission as a proposed assignment supported by several independent observations: EDS measurements reveal a deficiency of Ag in the synthesized powders (Tables 2 and 3); XPS measurements indicate electron-density redistribution involving Ag atoms (Figures 6 and S9); the excitation bands observed near 270–370 nm closely resemble the A-, B-, and C-bands previously reported for Ag-related centers in alkali halides (Figure 8a), and, finally, similar orange-red emission bands have been reported for Ag-centered defect states in related materials.^21–24^ Recent investigations^25,30^ have highlighted the important role of defect states and dopant-induced luminescence channels in determining the Vis emission properties of HDPs.

**Figure 11:**
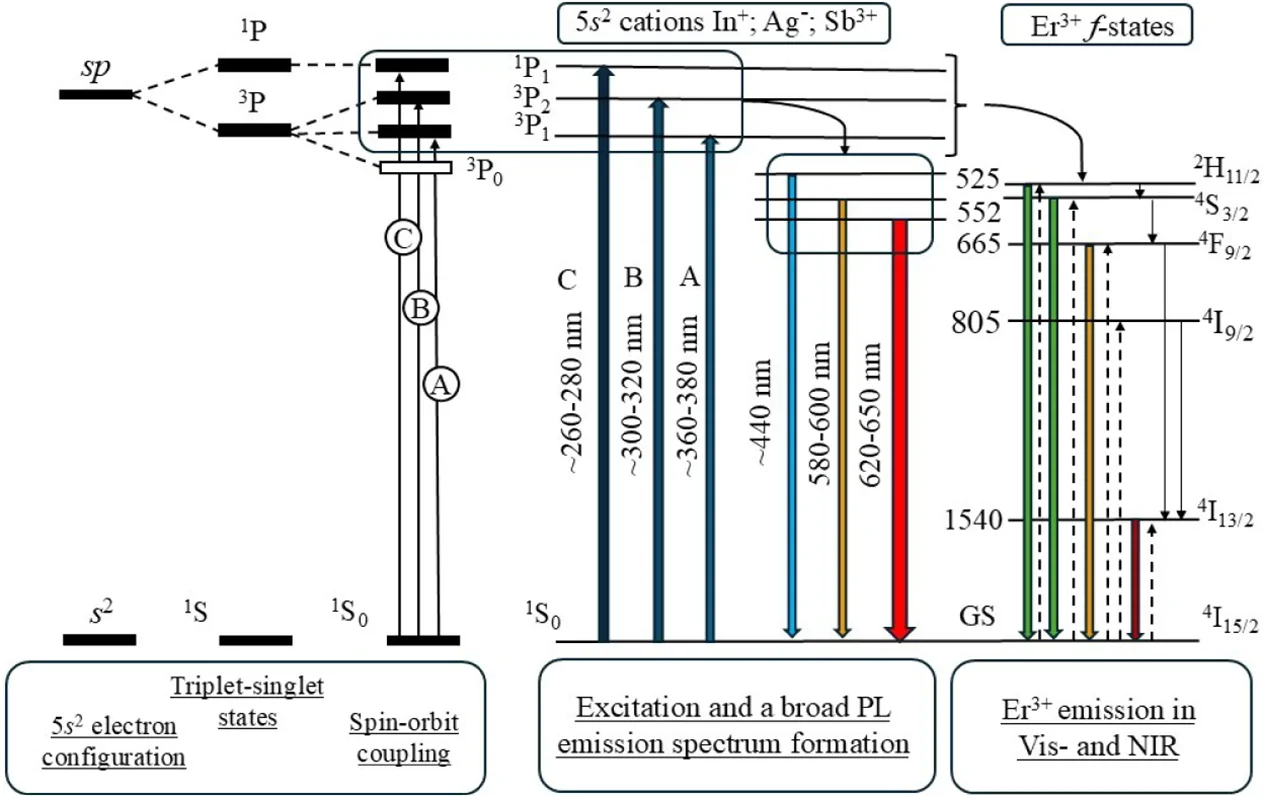
A scheme of A, B, and C bands, excitation, and PL spectra formation in Cs_2_AgInCl_6_ and the mechanism of energy transfer to Er^3+^ electrons with the following appearance of Er^3+^ luminescence because of *f-f* transitions.

The excitations form broad PL emission spectra from 400–800 nm because of the emission from Ag^–^, In^+^, and Sb^3+^ dopant ions. Part of the energy is transferred to the Er^3+^ dopant ions, which give rise to narrowband emission of Er^3+^ ions at ∼ 530 (^2^H_11/2_ → ^4^I_15/2_), 550 (^4^S_3/2_ → ^4^I_15/2_), 660 (^4^F_9/2_ → ^4^I_15/2_), 1510–1520 nm, 1547 nm, and 1562 nm. This corresponds to the ^4^I_13/2_ → ^4^I_15/2_ *f*-*f* transitions.^31,47,48^ Furthermore, recent studies on RE-doped double perovskites^42,49,50^ demonstrated efficient dopant-related Vis–NIR emission and host-to-RE energy transfer, which agrees well with the Er^3+^ luminescence observed in the present work.

This mechanism helps to obtain the PL emission in Sb-doped Cs_2_AgInCl_6_ and Er-doped Cs_2_AgInCl_6_ to cover a wide range from 440 to 1600 nm. We do not exclude the influence of STEs at low temperatures on the PL properties of Cs_2_AgInCl_6_, but this factor does not play a crucial role at room temperature.

### 3.7 DFT Results and Electronic Structure Discussion

Within the framework of density functional theory (DFT), quantum chemical simulations were performed to investigate Cs_2_AgInCl_6_, defect-containing Cs_2_AgInCl_6_-based crystal structures, and structures with Er^3+^ and Sb^3+^ dopants (see the SI). The optimized lattice parameters and corresponding energies per atom for all modeled structures are summarized in Table 4. Initially, Cs_2_AgInCl_6_ adopts the cubic structure (Sp.Gr. *Fm*3̅*m*) with a lattice parameter *a* = 10.4806 Å, which agrees with the data in Table 1. When the geometry is optimized, the structure remains cubic (Sp.Gr. *Fm*3̅*m*) with a slightly reduced lattice parameter *a* = 10.4132 Å. These data confirm that the chosen computational method accurately reproduces the geometric characteristics of the Cs_2_AgInCl_6_ lattice.

**Table 4:** Structural Parameters and the Total Energy of the Atom Systems Based on the Cs_2_AgInCl_6_ Crystal Structure Calculated with the DFT/PBE Method

Material	Lattice parameters *a* **=** *b* **=** *c* [Å]	Total energy [eV]	Total energy/atom [eV]
Cs_2_AgInCl_6_	10.4132	8140	203.50
Cs_2_AgInCl_6_+V_Cl_^–^	10.4223	8143	208.79
Cs_2_AgInCl_6_+V_Ag_^+^	10.4012	7249	185.87
Cs_2_AgInCl_6_+V_In_^3+^	10.3943	6974	178.82
Cs_2_AgInCl_6_+Er^3+^	10.4805	7248	181.20
Cs_2_AgInCl_6_+Sb^3+^	10.6535	6988	174.70
Cs_2_InCl_5_·H_2_O	*a* = 13.9959, *b* = 10.5096, *c* = 7.2972	128.55	3.57

Doping of Cs_2_AgInCl_6_ with trivalent ions, such as Sb^3+^ or Er^3+^ significantly influences both the structural and electronic properties of the host crystal. The incorporation of these dopants induces subtle lattice distortions and modifies the band structure, potentially benefiting various optoelectronic and luminescence applications. Substitution of In^3+^ with Sb^3+^ preserves the overall symmetry of the crystal but results in a slight increase in the lattice parameters and a reduction in the total energy per atom (Table 4), which increases the structural stability of the Sb-doped samples (as supported by the twofold higher yield of the precipitates; see Table S1).

In the case of Er^3+^ substitution, the crystal structure remains cubic but with a primitive unit cell and a statistical cation distribution (Sp.Gr. *Pm*3̅*m*). Nevertheless, the total energy per atom is reduced (Table 4), which also increases the stability of the Er-doped samples and explains the higher yield of the Er-doped precipitates, but only for 1 at. % and 3 at. % of the ErCl_3_ dopant (Table S1).

Moreover, the influence of defects was evaluated by introducing vacancies of Cl­(V_Cl_^–^), Ag­(V_Ag_^+^), and In­(V_In_^3+^). The V_Cl_^–^ defects were found to enlarge the lattice parameters, decreasing the stability of the crystal, whereas the V_Ag_^+^ and V_In_^3+^ defects decrease the unit cell. Additionally, V_Ag_^+^ and V_In_^3+^ vacancies reduce the total energy and enhance structural stability. The V_In_^3+^ and V_Ag_^+^ vacancies have a stabilizing effect, whereas V_Cl_^–^ has a destabilizing effect. Nevertheless, the lowest total energy per atom for Cs_2_InCl_5_·H_2_O (3.57 eV; Table 4) explains the low tendency to combine with AgCl, which increases the total energy of the system (203.5 eV for Cs_2_AgInCl_6_). Therefore, this factor can be decisive despite the opposite effects of V_Cl_^–^ and V_Ag_^+^ defects on the stability of the structure. This explains the absence of Ag and Cl in the structure, which determines the peculiarities of the optical properties of the samples (see Sections 3.5 and 3.6).

The electronic properties of the optimized Cs_2_AgInCl_6_ structure were calculated, and the results are presented in Table 5. All calculations were performed in the spin-restricted approach, but for the pristine Cs_2_AgInCl_6_ crystal, spin-up and spin-down calculations yield the same results (Figure 12). Cs_2_AgInCl_6_ is a direct-band gap semiconductor with a band gap of 4.42 eV at the Γ point. The Cl 3*p* electrons constitute the valence band (VB), and the conduction band (CB) is a hybrid of the Cs 5*d*, Cs 6*s*, and Cl 3*s* electrons, with its minimum (CBM) composed of In 5*s* electrons.

**Table 5:** Energy Gap Values and Nature Calculated by the DFT/PBE Method for the Cs_2_AgInCl_6_ Based Crystal Structures

Material	E_g_ [eV]			Nature of the energy band gap	
Cs_2_AgInCl_6_	4.42↑↓			Γ → Γ ↑↓	
Cs_2_AgInCl_6_+V_Cl_^–^	3.31↑		3.93↓	Γ → Γ↑	Γ → Γ↓
Cs_2_AgInCl_6_+V_Ag_^+^	4.51↑↓			Γ → Γ ↑↓	
Cs_2_AgInCl_6_+V_In_^3+^	4.59↑↓			Γ → Γ↑↓	
Cs_2_AgInCl_6_+Er^3+^	3.97↑↓			Γ → Γ↑↓	
Cs_2_AgInCl_6_+Sb^3+^	3.41↑↓			L → L↑↓	
Cs_2_InCl_5_·H_2_O	3.77↑	3.29↓		Γ → Γ↑↓	

**Figure 12:**
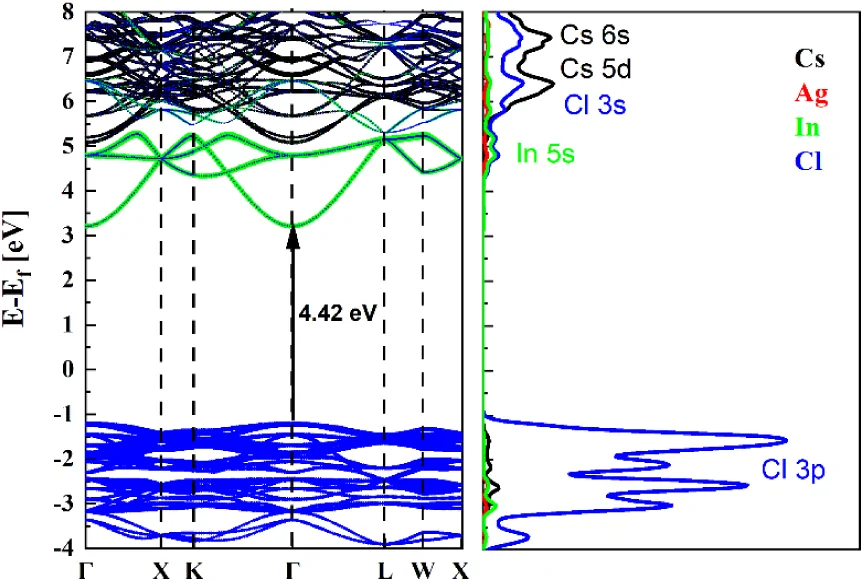
Energy band structure calculated by the DFT/PBE method for the pristine Cs_2_AgInCl_6_ crystal.

The V_Cl_^–^ vacancy with spin-up and spin-down exhibits a donor-trapping level composed of Cl 3*s* and In 5*s* electrons. For the spin-down structure, the energy dispersion of the trapping level is more significant than for spin-up (Figure 13a,b). The V_Ag_^+^ and V_In_^3+^ defective Cs_2_AgInCl_6_ crystals have an energy band structure very similar to the pristine structure, but with a slightly broader energy gap (Figure 13c,d).

**Figure 13:**
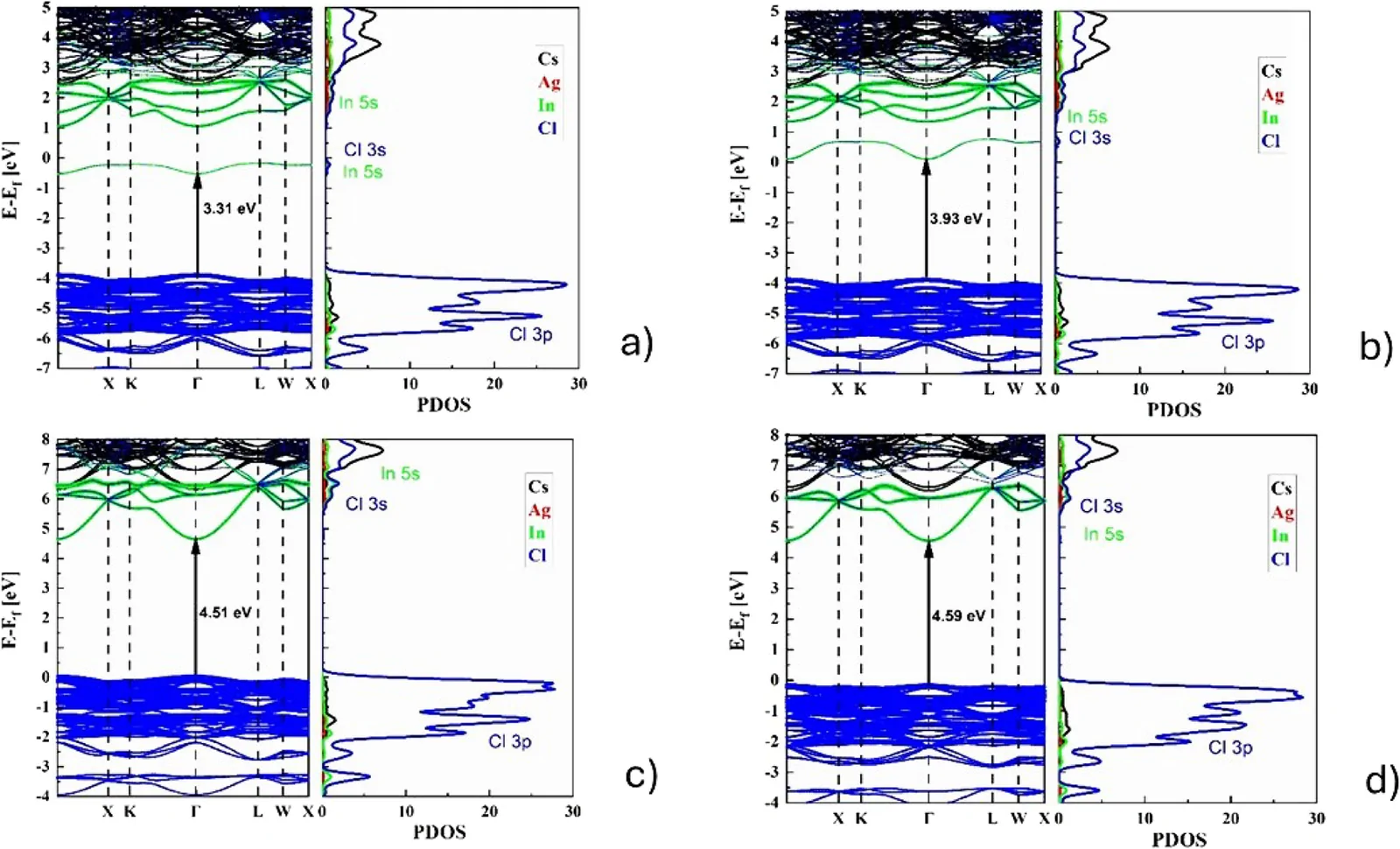
Energy band structure calculated by the DFT/PBE method for the defective Cs_2_AgInCl_6_ crystal with V_Cl_^–^ taking into account spin-up (a) and spin-down (b), with V_Ag_^+^—spin-up (c), and with V_In_^3+^—spin-up (d). Spin-up and spin-down for the Cs_2_AgInCl_6_ crystal with V_Ag_^+^ and V_In_^3+^ give the same results.

The Sb^3+^ and Er^3+^ dopants lead to band gap narrowing, shifting the Cl 3*s* and In 5*s* bands to lower energy. At the same time, Er^3+^ introduces an energy band between the Cl 3*s* and In 5*s* levels (see Figure 14a). Sb^3+^ adds an energy level below the Cl 3*s* and In 5*s* bands, creating a donor trapping state due to the Sb 5*p* electrons and an acceptor trapping level due to the Sb 5*s* electrons (see Figure 14b). This effect is attributed to the hybridization of Sb 5*s* ^2^ lone-pair electrons with Cl 3*p* orbitals, modifying the valence band edge and enhancing light absorption in the visible region, in agreement with the experimental absorption spectra (see Figure 7d). The band gap narrows to 3.41 eV, with electronic transitions occurring along the L → L direction. Er^3+^ doping results in the formation of sharp *f*–*f* transitions within the band gap and decreases the band gap to 3.97 eV. These results agree with the absorption spectra in Figure 7d.

**Figure 14:**
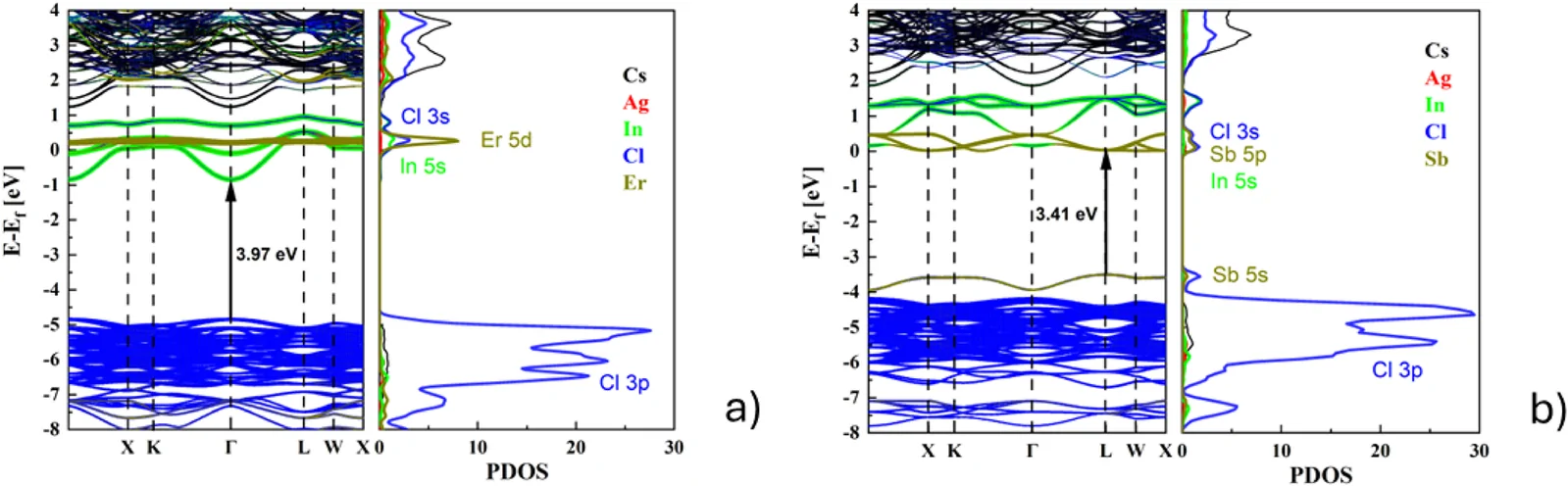
Energy band structure calculated by the GGA/PBE method for the Cs_2_AgInCl_6_ crystal doped with Er^3+^ with spin-up (a) and for the Cs_2_AgInCl_6_ crystal doped with Sb^3+^ with spin-up (b). Spin-down calculations give the same results.

For the defect structure Cs_2_InCl_5_·H_2_O, the band-gap structures with spin-up (Figure 15a) and spin-down (Figure 15b) approaches were calculated, and the top of the VB is created by electrons of 3*p* Cl and 2*p* O. The VB is formed by In 5*s* and Cl 3*p* electrons, which form a separate energy level in the band gap, and this affects the lower energy E_g_ of Cs_2_InCl_5_·H_2_O (Table 5).

**Figure 15:**
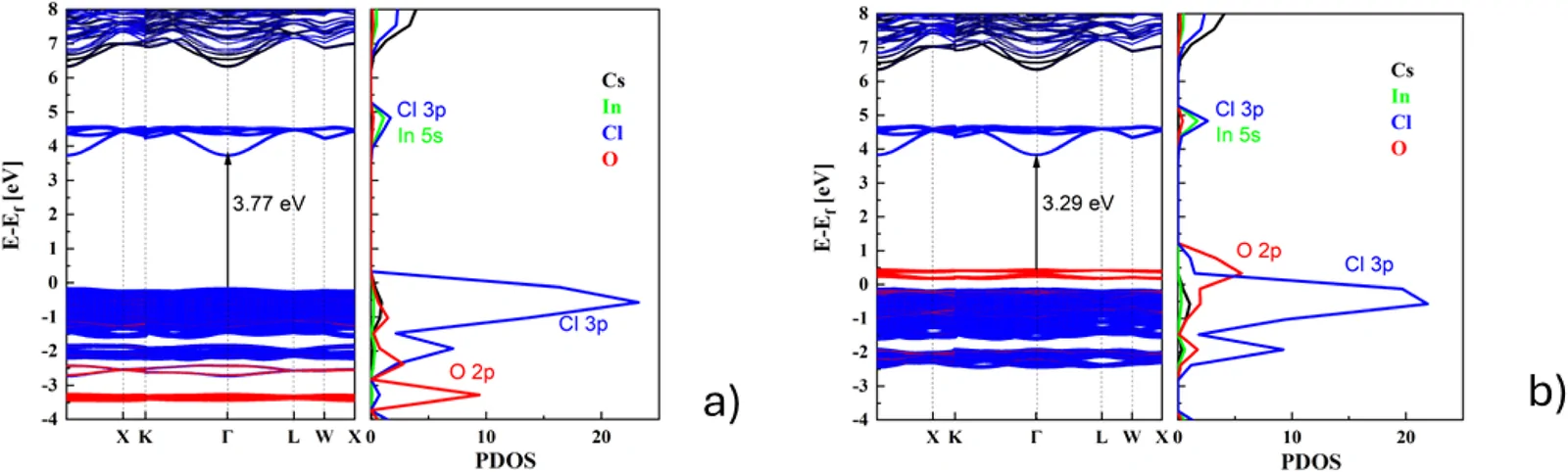
Energy band structure calculated by the GGA/PBE method for the Cs_2_InCl_5_·H_2_O crystal with spin-up (a) and spin-down (b) calculations.

The experimental band gap of Cs_2_AgInCl_6_ (4.29 eV) is compared directly with the calculated value of 4.42 eV; DFT predicts that Cl vacancies introduce trap states and reduce the band gap, consistent with the experimentally observed sub-band gap absorption features; Sb^3+^ doping is predicted to narrow the gap to approximately 3.4 eV through Sb-derived electronic states, which agrees with the observed red shift of the absorption edge; Er^3+^ doping introduces localized electronic states within the gap and provides channels for Vis and NIR luminescence; the experimentally observed excitation and emission spectra are discussed together with the calculated density of states and band structure results.

## 4 Conclusion

In this work, the structural, optical, and electronic properties of Cs_2_AgInCl_6_ and Sb^3+^- or Er^3+^-doped Cs_2_AgInCl_6_ microcrystals were investigated using XRD, Raman spectroscopy, XPS, UV–vis absorption, PL measurements, and DFT calculations. The experimental results show that the synthesized materials contain Ag and Cl deficiencies, which are attributed to incomplete AgCl incorporation during crystal growth. The formation of the intermediate Cs_2_InCl_5_·H_2_O phase and its high structural stability provide a possible explanation for the limited incorporation efficiency of AgCl in the precipitation-based synthesis route.

The optical properties of Cs_2_AgInCl_6_ are strongly influenced by defects and dopant-induced electronic states. Undoped Cs_2_AgInCl_6_ exhibits broad photoluminescence with dominant emission near 630 nm and weaker bands around 440 and 610 nm. Based on the combined analysis of excitation spectra, XPS results, defect chemistry, and literature data, these emissions are tentatively attributed to defect-related electronic states involving In^+^-, Ag^–^-, and vacancy-associated centers. The presence of chlorine vacancies may promote the formation of V_k_-type hole-trapping centers and other localized defect states that influence charge-carrier recombination processes. Although STEs may contribute to luminescence, the present results indicate that defect-related states play an important role in the room-temperature emission behavior of the studied samples. Defects may alter the local electronic structure of Cs_2_AgInCl_6_, potentially promoting the formation of Ag^0^, Ag^–^, In^+^, and Cl_2_^–^ -related defect states. While the XPS results indicate charge redistribution around the Ag and In ions, they do not provide direct proof of specific defect species. Therefore, the assignments of V_k_ centers, F-centers, and Ag- and In-related trapping states should be considered part of a proposed defect model, based on the combined evidence from EDS analysis, showing Ag and Cl deficiencies, XPS measurements, indicating charge redistribution around Ag and In ions, optical absorption, excitation, and PL spectra, DFT calculations of vacancy-induced electronic states, and comparison with previously reported defect chemistry of HDPs.

Sb^3+^ incorporation preserves the ordered cubic double perovskite structure and results in only weak photoluminescence enhancement. Unlike Sb-doped Cs_2_NaInCl_6_, no intense blue Sb-centered emission was observed, suggesting that the local crystal environment and defect structure of Cs_2_AgInCl_6_ did not favor efficient Sb^3+^-activated radiative recombination. Nevertheless, Sb incorporation improves sample stability and leads to significant band gap narrowing.

Er doping results in bands at 530, 550, 660, and 1520 nm that cover the visible and near-infrared regions ; however, their intensities are low. The Er-doped samples crystallize in a primitive cubic cell (Sp.Gr. *Pm*3̅*m*) with a statistical distribution of the cations.

Based on the DFT calculations, the band gap of Cs_2_AgInCl_6_ is 4.42 eV and direct, which agrees with our experimental data (E_g_ = 4.29 eV). However, Cl vacancies (V_Cl_^–^) create additional levels in the band gap, decreasing the value to 3.31 eV (spin up) and 3.93 eV (spin down). Ag and In vacancies (V_Ag_^+^; V_In_^3+^) mostly do not change the band gap values—4.51 eV for Ag vacancies and 4.59 eV for In vacancies—leaving the band gap direct. Sb doping decreases the band gap of the samples to 3.45 eV, which agrees well with E_g_ = 3.79 eV obtained from the absorption spectrum, and enhances light absorption in the visible region, which is particularly beneficial for applications such as photodetectors, photovoltaics, or photocatalysis. Er doping decreases the band gap to 3.97 eV (direct band gap) and forms sharp *f*–*f* transitions within the band gap.

Overall, the results reveal that the optical response of Cs_2_AgInCl_6_ is governed by a complex interplay among intrinsic defects, dopant ions, and host-lattice electronic states. While these defect-related processes may limit photoluminescence efficiency through nonradiative recombination pathways, they also enable band gap engineering and broadband Vis-to-NIR emission. Therefore, doped Cs_2_AgInCl_6_ double perovskites remain promising materials for photodetection, LEDs, lasers, bioimaging materials, and other optoelectronic applications.

## Supplementary Material



## Notes

Energy level diagram for an impurity ion with the n*s* ^2^ ground-state configuration (Figure S1); schematic of the synthesis technique of the halide double perovskites Cs_2_AgInCl_6_ and obtained precipitates under daylight and 254 nm light (Figures S2, S3); amount of chlorides used for the sample syntheses (Table S1); details of the refinement of the structures of the Cs_2_AgInCl_6_ single crystals, atomic coordinates, anisotropic parameters, interatomic distances, and coordination numbers of the atoms (Tables S2–S5); structural parameters obtained from Rietveld refinement of Er-doped Cs_2_AgInCl_6_ powder samples, atomic coordinates, site occupancies, and isotropic displacement parameters (Tables S6–S8); crystallographic data and structural parameters for Cs_2_InCl_5_·H_2_O single crystal and Cs_2_InBr_5_·H_2_O powder samples (Tables S9–S15); XRD diffractograms of Cs_2_AgInCl_6_, Sb (Er)-doped samples, and Cs_2_InBr_5_·H_2_O powder after Rietveld refinement. (Figures S4, S5, S8); SEM images and elemental mapping of selected microcrystals and powders (Figures S6, S7); XPS spectra and core-level binding energies for Cs_2_AgIn_0.9_Sb_0.1_Cl_6_ (Figure S9, Table S16); and photoluminescence spectra of undoped, Er^3+^ - and Sb^3+^ -doped Cs_2_AgInCl_6_ samples (Figure S10) (PDF)
