# Supplementary material for: Defects, (Sb, Er) Dopant Interactions, and Band Gap Engineering in Cs2AgInCl6 Double Perovskites
Source: J Phys Chem C Nanomater Interfaces. 2026 Sep 28;130(40):13774–85. doi: 10.1021/acs.jpcc.6c03164 (PMC13660115; doi:10.1021/acs.jpcc.6c03164)
Supplement: Supplementary file 1 [file jp6c03164_si_001.pdf]

Defect, (Sb, Er) Dopant Interactions and Band Gap Engineering in $Cs_2AgInCl_6$ Double Perovskites

Inna A. Ivashchenko[1], Wojciech Piotrowski[2], Marcin Nyk[2], Lubomir D. Gulay[3], Volodymyr O. Yukhymchuk[4], Galina Gurieva[5], Andreas Eich[5], Susan Schorr[5,6], Beata Szreniawa[1], Oleg Yu. Khyzhun[7,9], Natalya Kurgan[8], Malgorzata Makowska-Janusik[9], Karina V. Lamonova[10] and Katarzyna Matras-Postołek[*1]

[1]*Cracow University of Technology, Faculty of Chemical Engineering and Technology, Warszawska St. 24, 31-155 Cracow, Poland*

[2]*Institute of Advanced Materials, Wroclaw University of Science and Technology, Wyb. Wyspianskiego 27, 50-370 Wroclaw, Poland*

[3]*Lesya Ukrainka Volyn National University, Voli Ave. 13, 43000 Lutsk, Ukraine*

[4]*V.E. Lahskaryov Institute of Semiconductor Physics, National Academy of Sciences, Prospekt Nauki 41, Kiev 03028, Ukraine*

[5]*Helmholtz-Zentrum Berlin für Materialien und Energie, Hahn-Meitner-Platz 1, 14-109, Berlin, Germany*

[6]*Institut of Geological Sciences, Freie Universität Berlin, Kaiserswerther Str. 16-18, Berlin, Germany*

[7]*Frantsevich Institute for Materials Science, National Academy of Sciences of Ukraine, O. Pritsaka (Krzhizhanovsky) str., 3, 03-142 Kyiv, Ukraine*

[8]*G.V. Kurdyumov Institute of Metal Physics, National Academy of Sciences of Ukraine, Vernadsky St. 36, 03-142 Kyiv, Ukraine*

[9]*Faculty of Science and Technology, Jan Długosz University, Al. Armii Krajowej 13/15,42-200 Czestochowa, Poland*

[10]*O. O. Galkin Donetsk Institute for Physics and Engineering, National Academy of Sciences of Ukraine, Nauky ave. 46, 03-028 Kyiv, Ukraine*

[*]*Email: prof. Katarzyna Matras-Postołek (k.matras@pk.edu.pl)*

Content:

Fig. S1. Energy level diagram for an impurity ion with the ns^2 ground-state configuration.

Fig. S2. Schematic of the synthesis technique of the halide double perovskites$Cs_2AgInCl_6$ and obtained precipitates under daylight and 254 nm light.

Fig. S3 Samples $Cs_2AgInCl_6$ doped with Er^{3+} after precipitation synthesis under day light.

Fig. S4 Experimental (dots), calculated (solid) and difference (bottom scale) diffractograms of the $Cs_2AgInCl_6$ and Sb-doped samples.

Fig. S5 Experimental (dots), calculated (solid) and difference (bottom scale) diffractograms of the $Cs_2AgInCl_6$ Er doped samples.

Fig. S6 Mapping of the $Cs_2AgIn_{0.9}Cl_6$ $0.1ErCl_3$ powder sample.

Fig. S7 SEM photos of (a) $Cs_2AgIn_{0.9}Cl_6$ $0.1ErCl_3$ single crystal; (b) $Cs_2InCl_5{\cdot}H_2O$ single crystal; (c) $Cs_2InBr_5{\cdot}H_2O$ powder; (d) Mapping of the $Cs_2InBr_5{\cdot}H_2O$ powders.

Fig. S8 Experimental (dots), calculated (solid) and difference (bottom scale) diffractograms of the $Cs_2InBr_5{\cdot}H_2O$ powder sample.

Fig. S9 The XPS core-level spectra of $Cs_2AgIn_{0.9}Sb_{0.1}Cl_6$: (a) Cs 3*d*, (b) In 3*d*, (c) O 1*s* + Sb 3*d*, (d) Ag 3*d*, (e) Cl 2*p*, and (f) valence-band region.

Fig. S10 The emission spectra obtained at λ_{exc} = 270 nm and 340 nm for: a) $Cs_2AgInCl_6$; (b) Er^{3+} doped; (c) Sb^{3+} doped samples.

Table S1. Amount of chlorides used for the sample synthesis.

Table S2. The crystallographic characteristics and the details of the refinement of the structures of the $Cs_2AgInCl_6$ single crystals in two different variants.

Table S3. Atomic coordinates in the structures of the $Cs_2AgInCl_6$ single crystal.

Table S4. Anisotropic parameters of the structures of the $Cs_2AgInCl_6$ single crystal.

Table S5. Interatomic distances (d, Å) and coordination numbers (C.N.) of the atoms in two different variants of the structure of the $Cs_2AgInCl_6$ single crystal.

Table S6. Structural parameters obtained from Rietveld refinement of Er-doped $Cs_2AgInCl_6$ powder samples.

Table S7. Atomic coordinates, site occupancies, and isotropic displacement parameters for Er-doped powder samples obtained from Rietveld refinement.

Table S8. Interatomic distances and coordination numbers (C.N.) of atoms in the structures of the Er-doped powder samples.

Table S9. The crystallographic characteristics and the details of the refinement of the structures of the $Cs_2InCl_5{\cdot}H_2O$ single crystal.

Table S10. Atomic coordinates, Wyckoff positions, and site occupancies in the crystal structure of $Cs_2InCl_5{\cdot}H_2O$ single crystal.

Table S11. Anisotropic displacement parameters obtained from single-crystal structure refinement of $Cs_2InCl_5{\cdot}H_2O$.

Table S12. Interatomic distances (d, Å) and coordination numbers (C.N.) of the atoms of the $Cs_2InCl_5{\cdot}H_2O$.

Table S13. Results of crystal structure refinement of the $Cs_2InBr_5{\cdot}H_2O$ powder.

Table S14. Refined coordinates of atoms and their isotropic thermal parameters of the $Cs_2InBr_5{\cdot}H_2O$ powder.

Table S15. Interatomic distances and coordination numbers (C.N.) of atoms in the compound structures of the $Cs_2InBr_5{\cdot}H_2O$ powder.

Table S16. Binding energy values of core-level electrons (uncertainty is ±0.08 eV) as evaluated from the present XPS measurements made for the $Cs_2AgIn_{0.9}Sb_{0.1}Cl_6$ sample.

1. Methods of Synthesis

The set of the samples $Cs_2AgInCl_6$, $Cs_2AgIn_{0.9}Sb_{0.1}Cl_6$, $Cs_2AgIn_{1-x}Cl_6$: xEr^{3+}; x = 0.1; 0.3; 0.5, were synthesised using precipitate or hydrothermal methods as powders in the microcrystalline range using purchased from Sigma Aldrich (USA) chlorides (CsCl (99.5 wt.%), AgCl (99.9 wt.%), $InCl_3$ (99.99 wt.%), $SbCl_3$ (99.95 wt.%), and $ErCl_3$ (99.9 wt.%). They were combined in stoichiometric ratios to create the complete set of samples for the research (refer to Table S1). At the first stage, 10 ml of 36% HCl solutioni were poured into 50 ml flasks with glass spherical condensers, then calculated masses of AgCl (or $ErCl_3$) were added (see Table S1) and stirred. Three solutions were prepared separately: solution A contained 1 mmol $SbCl_3$ and 10 ml of 36% HCl; solution B consisted of 4.5 mmol $InCl_3$ with 10 ml of 36% HCl; and solution C was made up of 10 mmol CsCl added into 5 ml of 36% HCl. The calculated amounts of solutions A and B were added into the 50 ml flasks. Then the mixtures were heated to 90 °C, and − once the AgCl was dissolved − 1 ml of solution C was added to each flask, and immediately, precipitates formed (Fig. S2). For precipitation reaction the synthesis was finished after 2 hours ($Cs_2AgInCl_6$, $Cs_2AgIn_{0.9}Sb_{0.1}Cl_6$) or 5 hours (Er-doped samples) of rotation of the mixture at 90°C. While in case of the hydrothermal method, the solutions were transferred to 100 ml Teflon containers and placed into a stainless steel reactor for hydrothermal synthesis. These were heated in a furnace to 120°C and annealed for 10 hours. After the furnace was

turned off, the vessels were slowly cooled to 30°C. The final precipitates were filtered and washed with anhydrous ethanol to remove residual acid from the crystal surfaces, then calcined for 2 hours in an oven at 160 °C to eliminate traces of acid and water (Figs. S2, S3). The same was done for powders obtained by precipitation method.

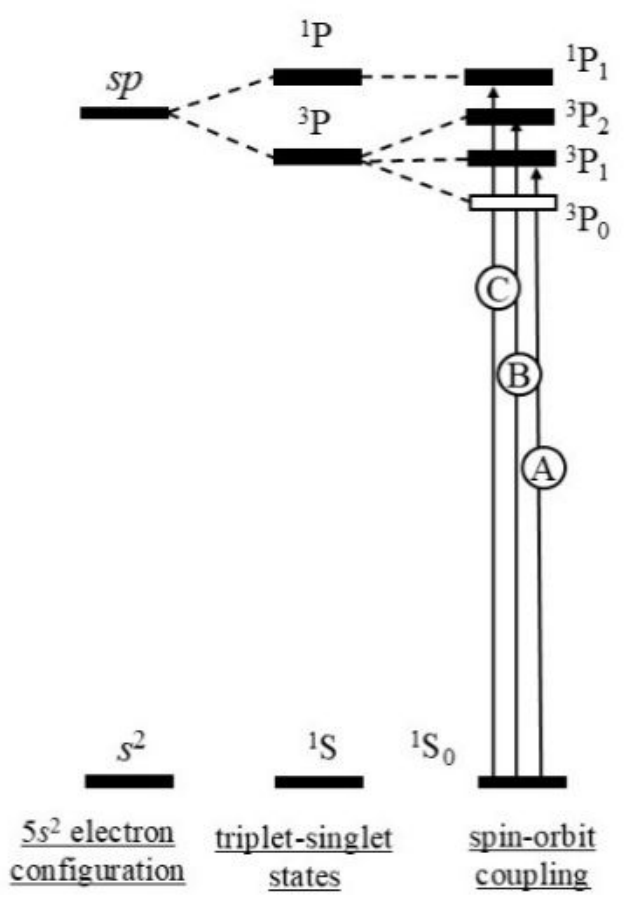

Fig. S1. Energy level diagram for an impurity ion with the ns^2 ground-state configuration [1].

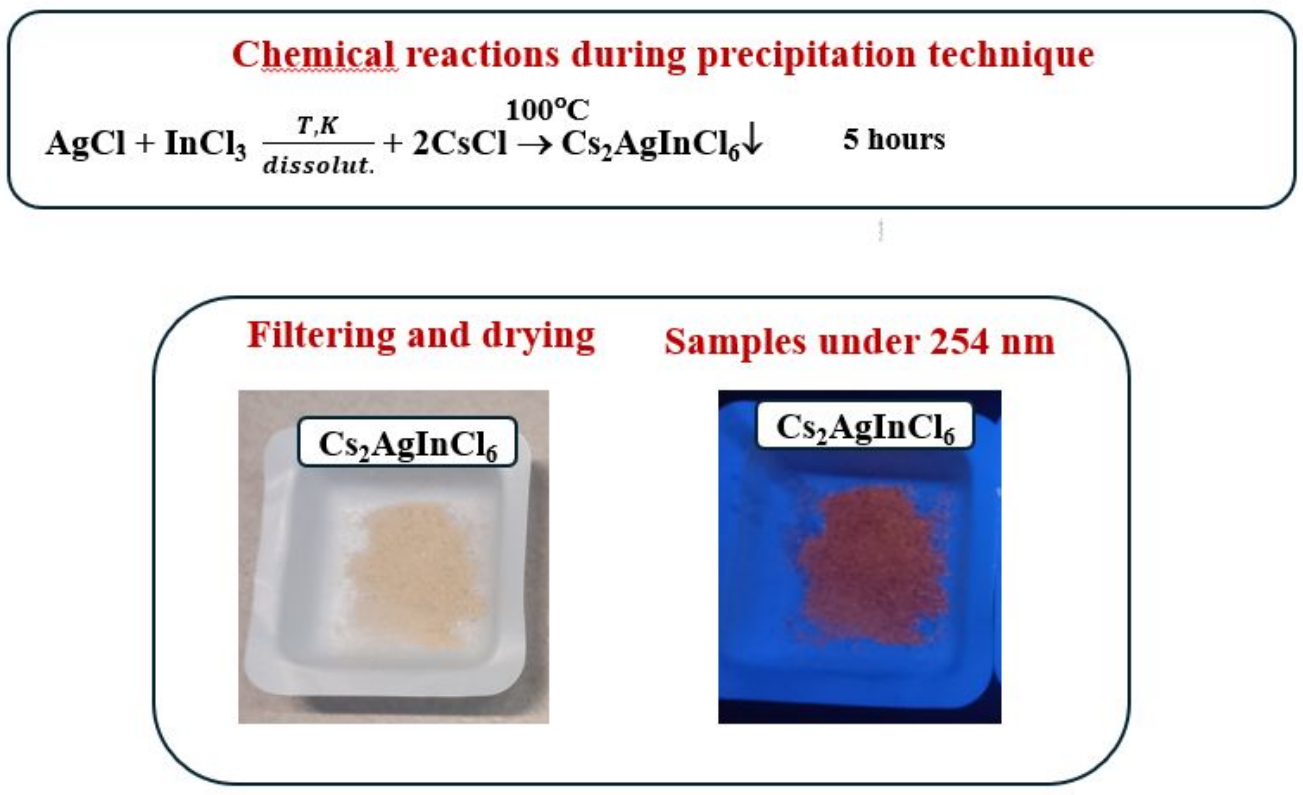

Fig. S2. Schematic of the synthesis technique of the halide double perovskites $Cs_2AgInCl_6$ and obtained precipitates under day light and 254 nm light.

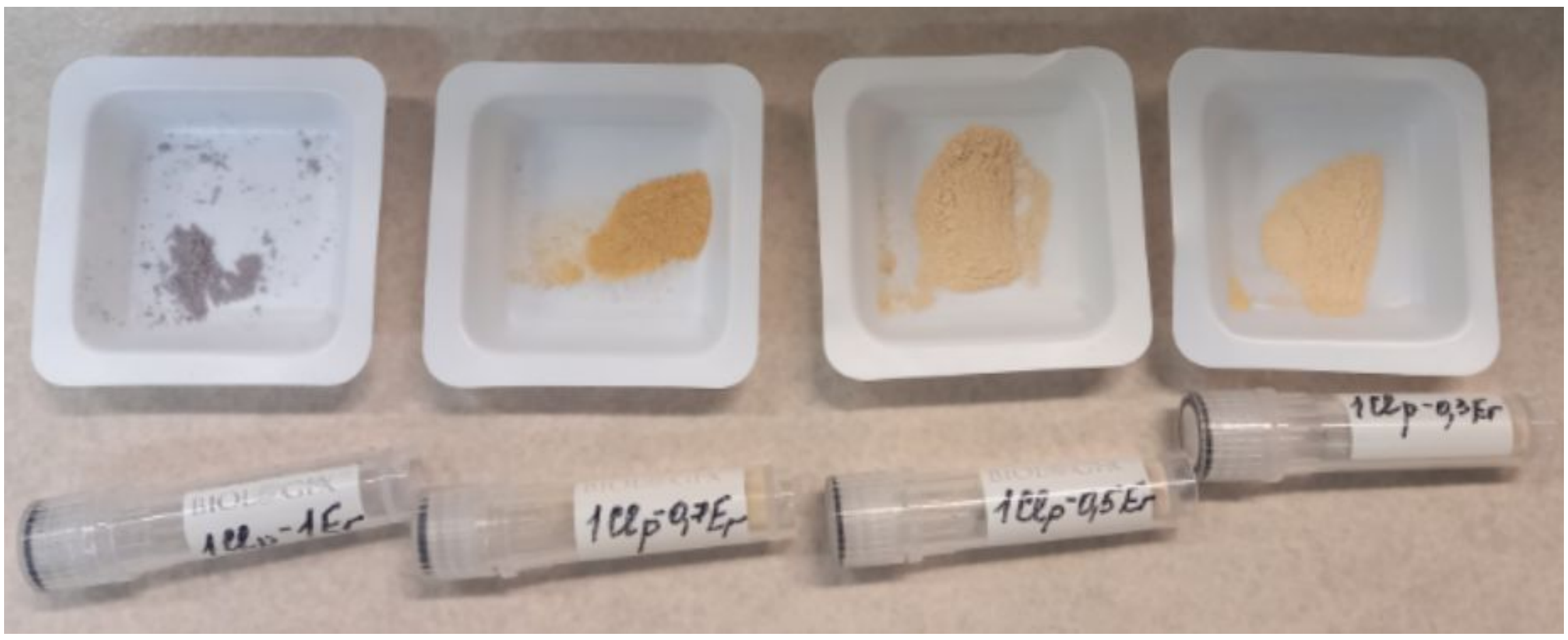

Fig. S3 Samples $Cs_2AgInCl_6$ doped with Er^{3+} after precipitation synthesis under day light.

Table S1. Amount of chlorides used for the sample syntheses

Composition	n, CsCl mmol	n, AgCl mmol	n, $InCl_3$ mmol	n, $SbCl_3$ mmol	n, $ErCl_3$ mmol	m_p, gr	η, % $m_p/m_{calc}\cdot100\%$
$Cs_2AgInCl_6$	2	1	1	-	-	0.23904	0.23904/0.70122·100%=34%
$Cs_2AgIn_{0.9}Cl_6\cdot0.1SbCl_3$	2	1	0.9	0.1	-	0.46161	0.46161/0.70191·100%=66%
$Cs_2AgIn_{0.9}Cl_6\cdot0.1ErCl_3$	2	1	0.9	-	0.1	0.42062	0.42062/0.7065·100%=60%
$Cs_2AgIn_{0.7}Cl_6\cdot0.3ErCl_3$	2	1	0.7	-	0.3	0.38740	0.38740/0.71697·100%=54%
$Cs_2AgIn_{0.5}Cl_6\cdot0.5ErCl_3$	2	1	0.5	-	0.5	0.20855	0.20855/0.73092·100%=29%

2. Characterization Methods

The obtained powders have been investigated with **X-ray powder diffraction (XRD)** (using the SmartLab powder diffractometer, CuKα radiation, 2θ-range of 10-120°, 0.05° scan step). The crystal structure of the samples was evaluated applying the powder method using the WinCSD software [2].

Single crystal investigations were performed using **single-crystal XRD**, parameters of the measurements are shown in the Table S2-S5, S10-S12. The crystal structures have been calculated with Patterson methods and refined using the full-matrix least-squares method using SHELXL-2018 program [3].

The HDPs crystal shapes and sizes were studied using an Apreo 2s LoVac (Thermo Fisher Scientific) scanning electron microscope. The element distribution was determined by means of **energy-dispersive X-ray spectroscopy (EDS)**.

For the registration of the **Raman signals**, an MDR-23 spectrometer equipped with a cooled CCD detector iDus 420 Andor (UK) was used. The laser power density on the sample surface was less than 10^3 W/cm^2, to preclude any thermal modification of the samples. The spectral resolution for all excitation radiation wavelengths did not exceed 4 cm^{-1} and was determined from the Si phonon peak width of a Si single crystal. The Si phonon peak position of 520.5 cm^{-1} was used as a reference for determining the position of the peaks in the Raman spectra acquired at different λ_{exc}.

The specimens were tested also employing possibilities of **X-ray photoelectron spectroscopy (XPS)**. Measurements of the XPS spectra of the samples under study were made using a Perkin Elmer spectrometer equipped with a monochromatized Al Kα source (E=1486.6

eV). Since the XPS method is known to be very sensitive to surface charging effects, they were compensated in the present work by using a special flood gun equipped with the spectrometer.

The **absorption spectra** were recorded on a Shimadzu UV-2600 UV-Vis spectrophotometer. A Hitachi F-4600 fluorescence spectrometer was used to measure the **emission and excitation spectra** of the samples. The n**ear-IR room-temperature emission spectra** of the samples were recorded using an NIRQuest 512-2.2 (Ocean Optics) fiber-coupled spectrophotometer, with excitation provided by a 976 nm continuous-wave (CW) laser diode (4W, Optel, Poland). To minimize the signal from scattered excitation laser light, a 1000 nm long-pass filter (Thorlabs) was used for near-infrared measurements, placed in front of the respective spectrometers.

3. DFT Calculations

Quantum chemical modeling was conducted to investigate both doped and defect-containing $Cs_2AgInCl_6$-based crystal structures, with Er^{3+} and Sb^{3+} incorporated as dopants. These simulations were performed within the framework of density functional theory (DFT), using the Vienna Ab initio Simulation Package (VASP). The projector augmented wave (PAW) method [4] was employed to describe the interaction between valence electrons and ionic cores. Exchange-correlation interactions were treated using the Perdew–Burke–Ernzerhof (PBE) [5] functional within the generalized gradient approximation (GGA). Spin-polarized sv-GW basis sets were used to appropriately model open-shell systems and potential magnetic effects. A plane-wave energy cutoff of 500 eV was applied to ensure reliable numerical accuracy. The Brillouin zone was sampled using a 6×6×6 Γ-centered Monkhorst–Pack grid [6]. The self-consistent field (SCF) cycles were converged to a strict threshold of 10^{-8} eV, ensuring precision in total energy and charge density distribution.

The study began with geometry optimization to identify the energetically most favorable structure. Full structural relaxations were carried out, allowing the optimization of atomic positions, unit cell volume, and lattice shape. Convergence criteria were set to 10^{-5} eV for total energy and 0.03 eV/Å for interatomic forces.

To evaluate the effect of dopants, supercells were constructed with Er^{3+} and Sb^{3+} ions replacing In^{3+} at a concentration of 2.5 at.%. These models were designed to mimic realistic doping scenarios and to assess their influence on structural and electronic properties. Each doped supercell was fully optimized to account for local distortions introduced by the dopants. The substitutions were treated as isovalent, and charge neutrality was preserved in all cases.

In addition, defective $Cs_2AgInCl_6$ structures were modeled by introducing vacancies of chlorine (V_{Cl}), silver (V_{Ag}), and indium (V_{In}). For each defect type, the respective atom was removed from the unit cell, and the resulting supercell was fully relaxed to capture the structural changes associated with the vacancy.

For analysis of electronic band structure, calculations were performed along a high-symmetry path in the Brillouin zone defined as: Γ–X–U–K–Γ–L–W–X, with high-symmetry points located at: X (0.500; 0.000; 0.500), U (0.625; 0.250; 0.625), K (0.375; 0.375; 0.750), L (0.500; 0.500; 0.500), and W (0.500; 0.250; 0.750).

Spin polarization was included in all calculations, and Fermi–Dirac smearing with a width of 0.05 eV was applied to aid convergence. A stringent threshold of 10^{-8} eV was used for the self-consistent field (SCF) calculations to ensure precision in energy and charge density evaluations.

Table S2. The crystallographic characteristics and the details of the refinement of the structures of the $Cs_2AgInCl_6$ single crystals in two different variants

Emperical formula	$Cs_2AgInCl_6$	$Cs_2AgInCl_6$
Formula weight	701.22	700.21
S.G.	$Pm\bar{3}m$ (№221)	$Fm\bar{3}m$ (№225)
Unit cell parameters, *Å*	*a*= 5.23373(7)	*a*= 10.4695(2)
V, $Å^3$	143.362(6)	1147.57(7)
Number of atoms in the unit cell; Z	5; 0.5	40; 4
Calculated density, g/sm^3	4.061	4.059
Absorbtion coefficient, mm^{-1}	11.306	11.300
Radiation type,wavelength, *Å*	Mo Kα, 0.71073	Mo Kα, 0.71073
Single-crystal diffractometer	Bruker AXS X8 APEX II	Bruker AXS X8 APEX II
F(000)	154	1232
Θ range for data collection	3.893 – 64.261	3.370 – 52.002
Range of indexes	$-13 \le h \le 13$; $-12 \le k \le 13$<br>$-13 \le l \le 13$	$-23 \le h \le 23$; $-23 \le k \le 23$<br>$-23 \le l \le 23$
Reflections collected	21925	49898
Independent reflections	297	389
Refinement method	Full-matrix least-square on F^2	Full-matrix least-square on F^2
Data/restraints/parameters	297/0/6	389 /0/8
Goodness-of-fit on F^2	1.208	1.165
R [$I>2\sigma(I)$]	$R1$= 0.0279, $wR2$= 0.0881	$R1$= 0.0178, $wR2$= 0.0381
R (all data)	$R1$= 0.0357, $wR2$= 0.0924	$R1$= 0.0285, $wR2$= 0.0427
Shape; colour	grown, natural faces;<br>transparent white	grown, natural faces;<br>transparent white
Crystal size, µm	160×200×160	160×200×160
Extinction coefficient	0.151(15)	0.00411(12)
The highest difference peak and deepest hole, $Å^{-3}$	1.496 and -2.217	1.060 and -0.926

Table S3. Atomic coordinates in the structures of the $Cs_2AgInCl_6$ single crystal

Atom	Position	*x/a*	*y/b*	*z/c*	Occ.
			$Sp. Gr. Pm\bar{3}m$		
Cs<br>M1<br>Cl	1*b*<br>1*a*<br>3*d*	1/2<br>0<br>1/2	1/2<br>0<br>0	1/2<br>0<br>0	1<br>0.5 Ag + 0.5 In<br>1
			$Sp. Gr. Fm\bar{3}m$		
Cs<br>Ag<br>In<br>Cl	8*c*<br>4*b*<br>4*a*<br>24*e*	1/4<br>1/2<br>0<br>0.23964(6)	1/4<br>1/2<br>0<br>0	1/4<br>1/2<br>0<br>0	1<br>1<br>1<br>1

Table S4. Anisotropic parameters of the structures of the $Cs_2AgInCl_6$ single crystal

Atom	$U_{eq.}$, $Å^2$	U_{11}	U_{22}	U_{33}	U_{23}	U_{13}	U_{12}
			$Sp. Gr. Pm\bar{3}m$				
Cs<br>M1<br>Cl	0.02792(14)<br>0.01734(12)<br>0.0329(3)	0.02792(14)<br>0.01734(12)<br>0.0281(5)	0.02792(14)<br>0.01734(12)<br>0.0353(4)	0.02792(14)<br>0.01734(12)<br>0.0353(4)	0<br>0<br>0	0<br>0<br>0	0<br>0<br>0
			$Sp. Gr. Fm\bar{3}m$				
Cs<br>Ag<br>In<br>Cl	0.02761(9)<br>0.02175(10)<br>0.01330(8)<br>0.02807(16)	0.02761(9)<br>0.02175(10)<br>0.01330(8)<br>0.0141(2)	0.02761(9)<br>0.02175(10)<br>0.01330(8)<br>0.0350(2)	0.02761(9)<br>0.02175(10)<br>0.01330(8)<br>0.0350(2)	0<br>0<br>0<br>0	0<br>0<br>0<br>0	0<br>0<br>0<br>0

$U_{eq.}$ is defined as one third of the trace of the orthogonalized U_{ij} tensor. The anisotropic temperature factor exponent takes the form: $-2\pi^2[h^2a^{*2}U_{11} + ... + 2hka^*b^*U_{12}]$

Table S5. Interatomic distances (d, Å) and coordination numbers (C.N.) of the atoms in two different variants of the structure of the $Cs_2AgInCl_6$ single crystal

$Sp. Gr. Pm\bar{3}m$				$Sp. Gr. Fm\bar{3}m$			
Atoms		d, Å	C.N.	Atoms		d, Å	C.N.
Cs	–12Cl	3.70081(5)	12	Cs	– 12Cl	3.70312(7)	12
∗M1	– 6Cl	2.61686(3)	6	Ag	– 6Cl	2.7258(6)	6
-	-	-	-	In	– 6Cl	2.5089(6)	6

∗M1= 50 % Ag+ 50 % In

Table S6. Structural parameters obtained from Rietveld refinement of Er-doped $Cs_2AgInCl_6$ powder samples

Composition	$Cs_2AgIn_{0.9}Cl_6$ **$0.1ErCl_3$**	$Cs_2AgIn_{0.7}Cl_6\cdot$**$0.3ErCl_3$**	$Cs_2AgIn_{0.5}Cl_6\cdot$**$0.5ErCl_3$**
	$CsAg_{0.5}In_{0.45}Cl_3$ $0.05ErCl_3$	$CsAg_{0.5}In_{0.35}Cl_3\cdot 0.15ErCl_3$	$CsAg_{0.5}In_{0.25}Cl_3\cdot 0.25ErCl_3$
Sp. Gr	$Pm\bar{3}m$	$Pm\bar{3}m$	$Pm\bar{3}m$
Unit cell parameters, Å	a= 5.24204(9)	a= 5.24234(6)	a= 5.24249(5)
V, Å^3	144.046(7)	144.071(5)	144.083(5)
Number of atoms in cell	5	5	5
Calculated density (g/cm^3)	4.0716(2)	4.1313(2)	4.1914(2)
Absorption coefficient (1/cm)	910.31	903.82	897.43
Radiation and wavelenght (Å)	CuKα; 1.54185	CuKα; 1.54185	CuKα; 1.54185
Diffractometer	SmartLab	SmartLab	SmartLab
Mode of refinement	Full profile	Full profile	Full profile
Number of free parameters	5	5	5
R_I; R_p	0.0899; 0.2504	0.0560; 0.2078	0.0622; 0.2071
Scale factor	0.4672(2)	0.5545(5)	0.5284(4)
Texture axis and parameter	[0 0 1] 1.0966	[0 0 1] 1.2548	[0 0 1] 1.2524

Table S7. Atomic coordinates, site occupancies, and isotropic displacement parameters for Er-doped powder samples obtained from Rietveld refinement

Atom	Wyckoff pos.	*x/a*	*y/b*	*z/c*	Occ.	$B_{isot.}$, Å^2		
**$CsAg_{0.5}In_{0.45}Er_{0.05}Cl_3$***								
Cs	1*b*	1/2	1/2	1/2	1	0.10(14)	1.80(15)*	1.64(13)**
M	1*a*	0	0	0	0.50 Ag + 0.45 In + 0.05 Er	0.38(15)		
Cl1	3*d*	1/2	0	0	1	1.4(3)		
**$CsAg_{0.5}In_{0.35}Er_{0.15}Cl_3$***								
M	1*a*	0	0	0	0.50 Ag + 0.35 In + 0.15 E	1.50(15)		
Cl1	3*d*	1/2	0	0	1	2.9(3)		
**$CsAg_{0.5}In_{0.25}Er_{0.25}Cl_3$***								
M	1*a*	0	0	0	0.50 Ag + 0.25 In + 0.25 Er	1.24(13)		
Cl1	3*d*	1/2	0	0	1	2.1(2)		

* the compositions were established based on crystal structure calculations by powder method

Table S8. Interatomic distances and coordination numbers (C.N.) of atoms in the structures of the Er-doped powder samples

Atoms	Interatomic distances, Å			C.N.
Atoms	**$CsAg_{0.5}In_{0.45}Er_{0.05}Cl_3$**	**$CsAg_{0.5}In_{0.35}Er_{0.15}Cl_3$**	**$CsAg_{0.5}In_{0.25}Er_{0.25}Cl_3$**	
M – 6Cl	2.6210(1)	2.6212(3)	2.6212(1)	6
Cs – 12Cl	3.7067(1)	3.7071(2)	3.7070(1)	12

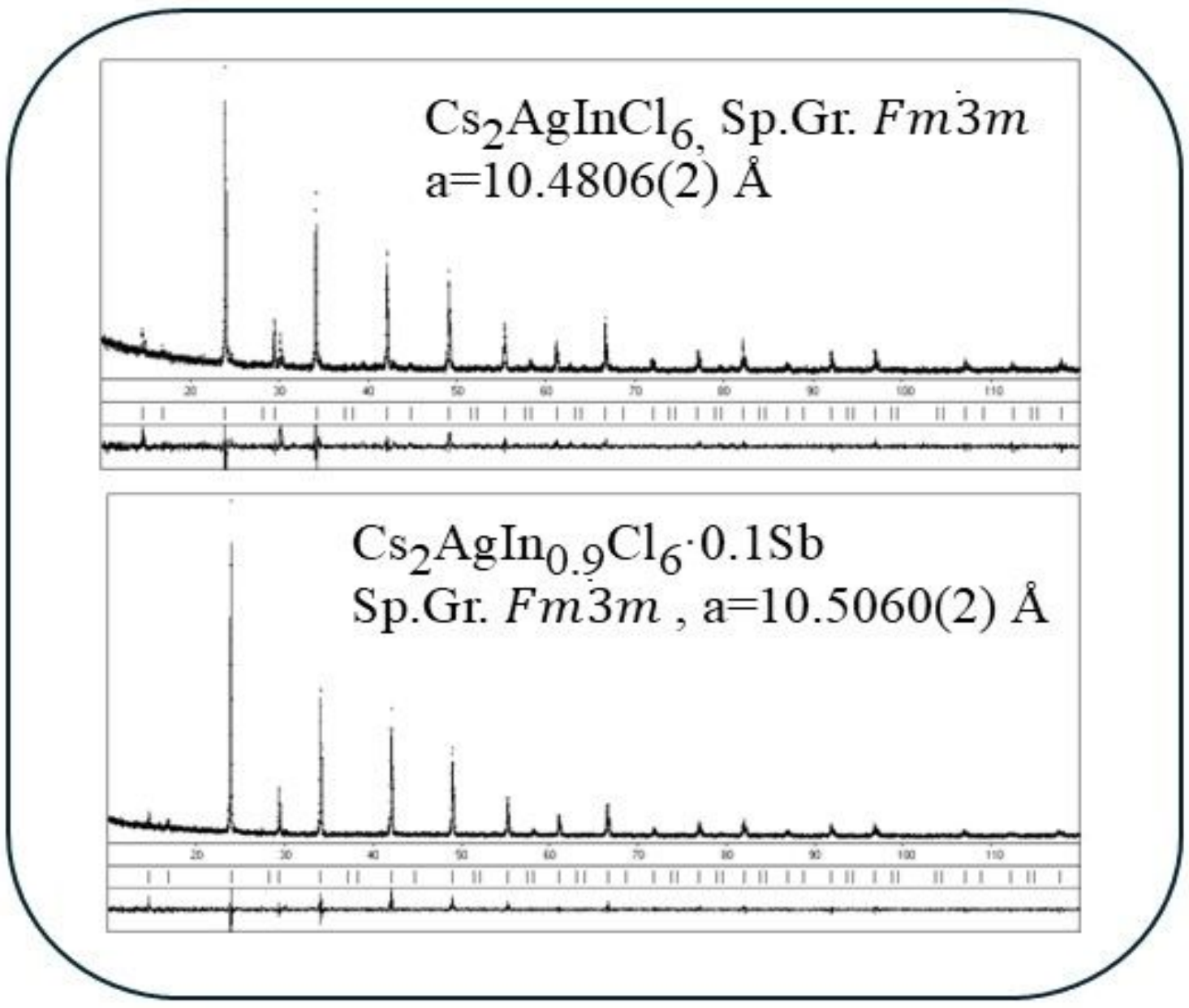

Fig. S4 Experimental (dots), calculated (solid) and difference (bottom scale) diffractograms of the $Cs_2AgInCl_6$ and Sb-doped samples**.**

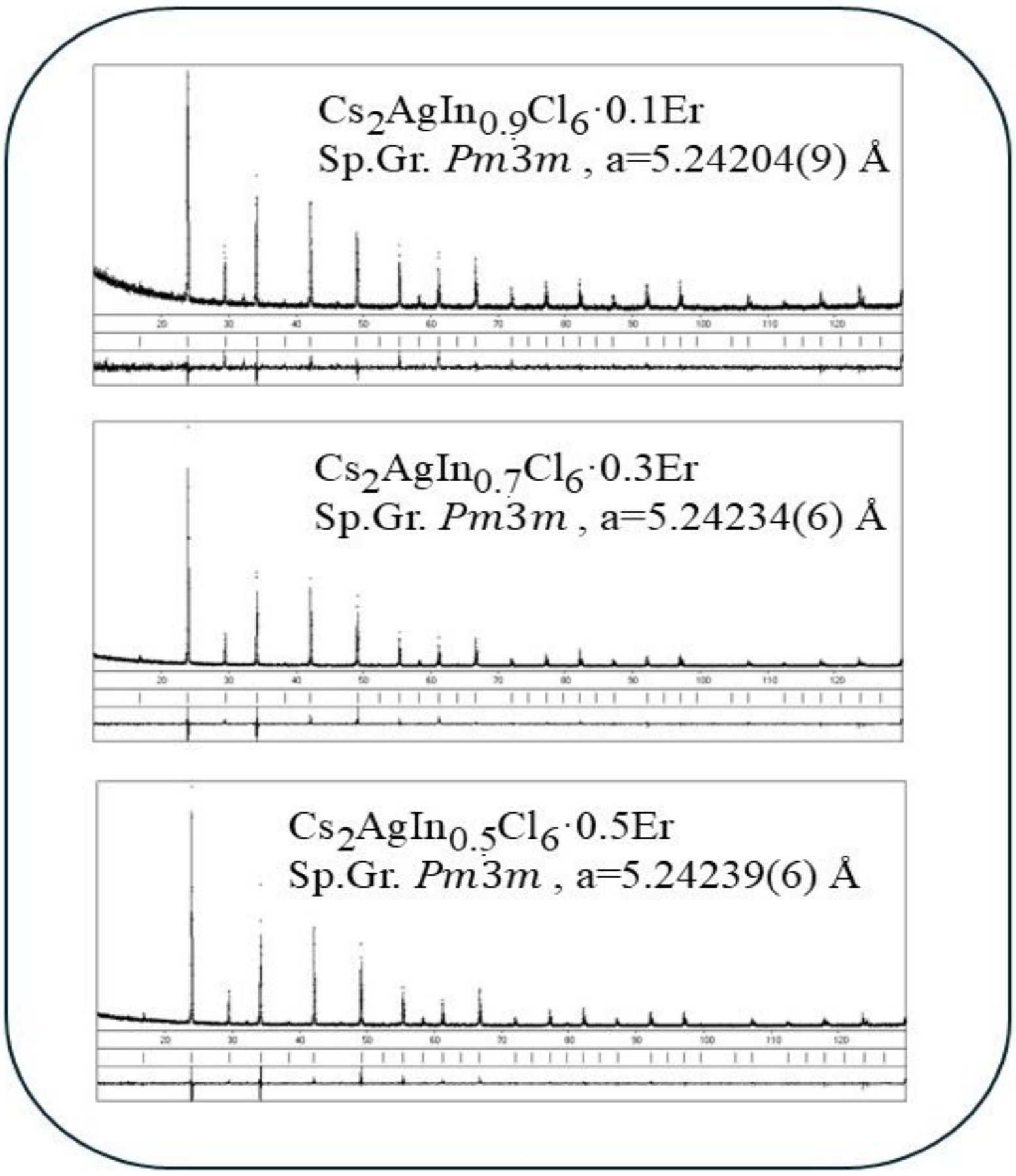

Fig. S5 Experimental (dots), calculated (solid) and difference (bottom scale) diffractograms of the Er doped $Cs_2AgInCl_6$ samples.

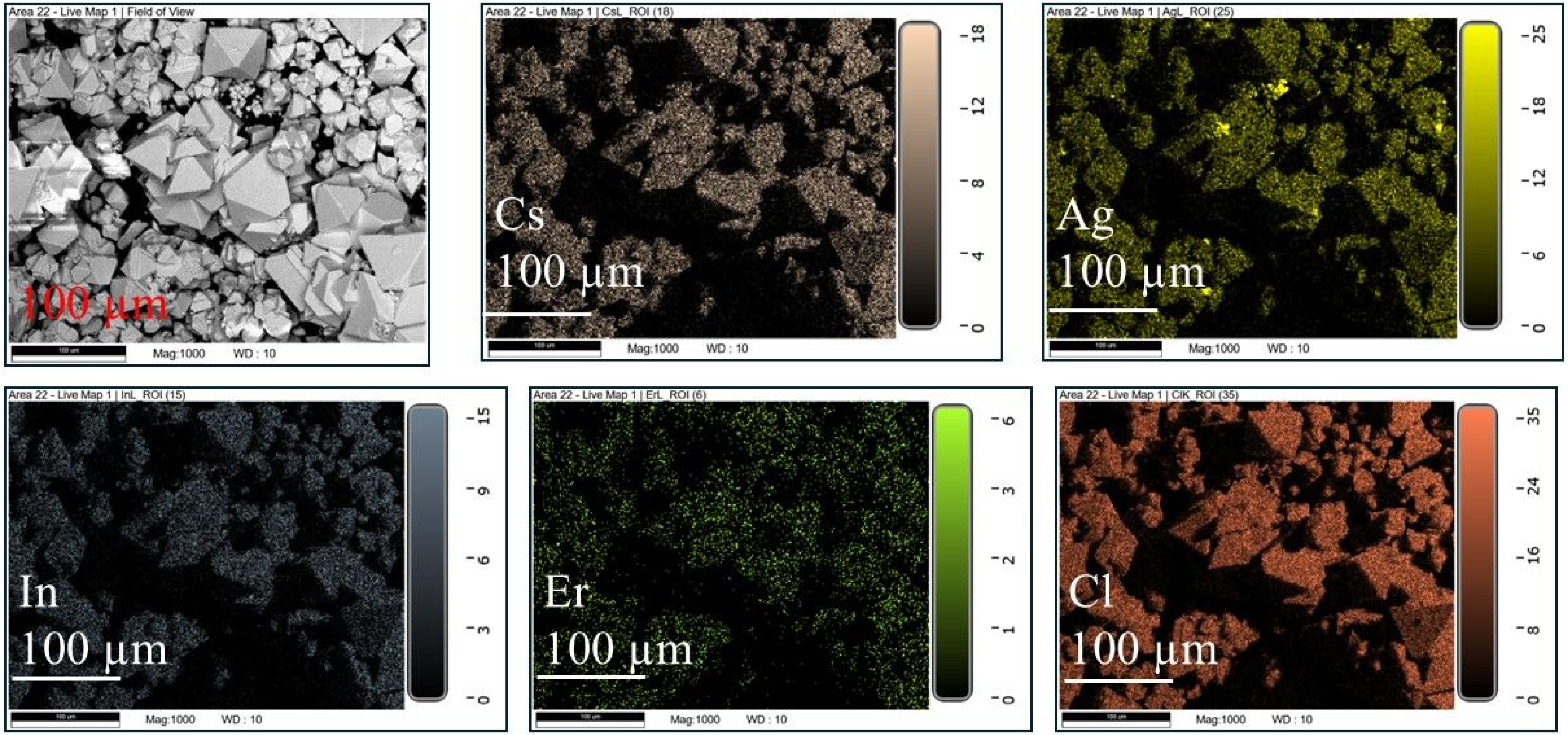

Fig. S6 Mapping of the $Cs_2AgIn_{0.9}Cl_6$ 0.1$ErCl_3$ powder sample .

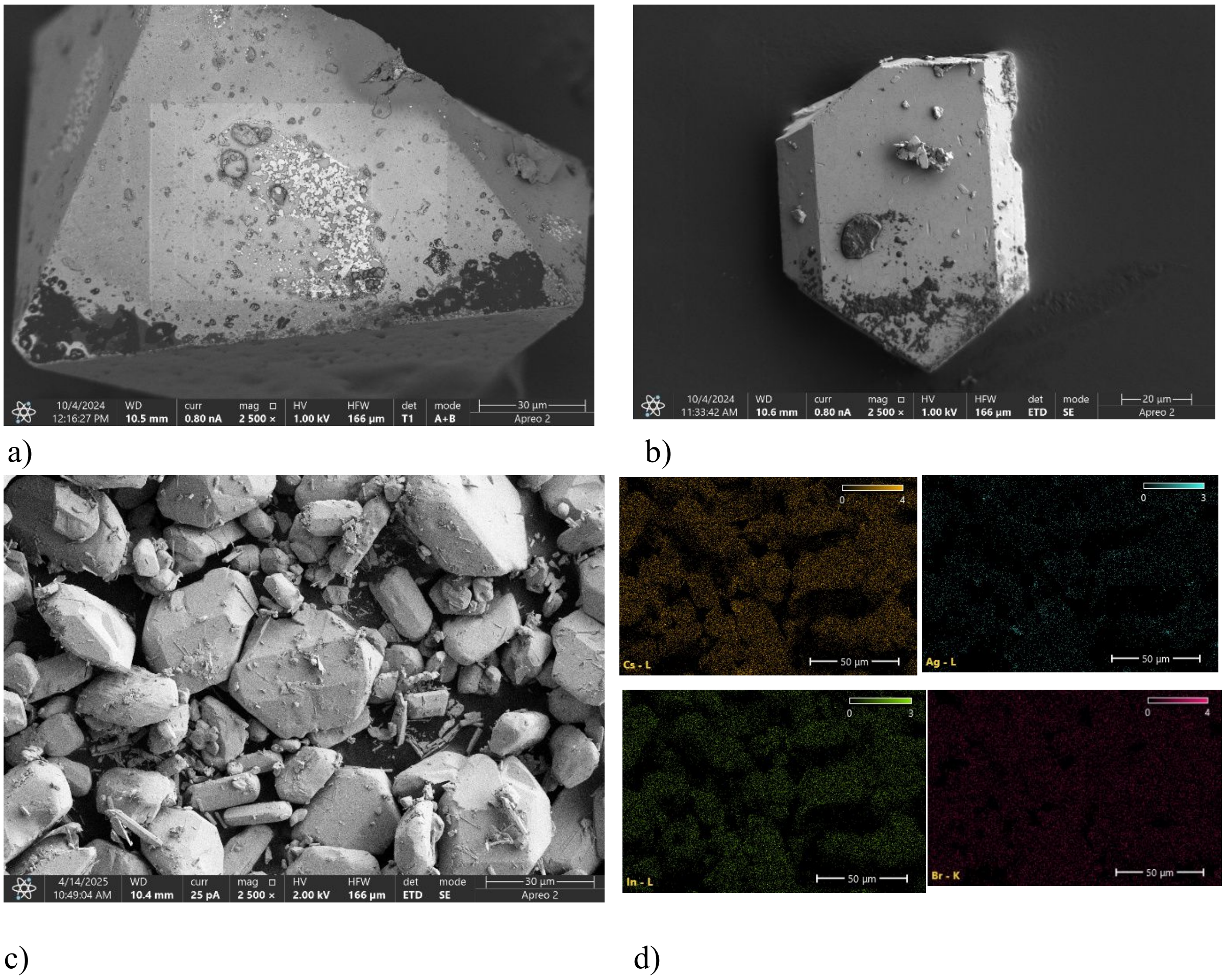

Fig. S7 SEM photos of (a) $Cs_2AgIn_{0.9}Cl_6$ 0.1$ErCl_3$ single crystal; (b) $Cs_2InCl_5 \cdot H_2O$ single crystal; (c) $Cs_2InBr_5 \cdot H_2O$ powder; (d) Mapping of the $Cs_2InBr_5 \cdot H_2O$ powder.

Table S9. The crystallographic characteristics and the details of the refinement of the structures of the $Cs_2InCl_5 \cdot H_2O$ single crystal

Emperical formula	$Cs_2InCl_5 \cdot H_2O$
Formula weight	573.89
S.G.	*Pnma* (№62)
Unit cell parameters, *Å*	*a* = 14.4034(4),<br>*b* = 10.3700(3), *c* = 7.4006(2)
V, $Å^3$	1105.38(5)
Z	4
Calculated density, g/sm^3	3.448
Absorbtion coefficient, mm^{-1}	9.775
Radiation type,wavelength, *Å*	Mo Kα, 0.71073
Single-crystal diffractometer	Bruker AXS X8 APEX II
F(000)	1008
Θ range for data collection	2.828 – 31.437
Range of indexes	$-21 \le h \le 21$; $-15 \le k \le 15$<br>$-10 \le l \le 10$
Reflections collected	73844
Independent reflections	1916
Refinement method	Full-matrix least-square on F^2
Data/restraints/parameters	1916/0/50
Goodness-of-fit on F^2	1.154
R [$I>2\sigma(I)$]	*R*1= 0.0310, *wR*2= 0.0913
R (all data)	*R*1= 0.0509, *wR*2= 0.1160
Shape; colour	grown, natural faces;<br>transparent white
Crystal size, µm	110×130×80
Extinction coefficient	0.0004(2)
The highest difference peak and deepest hole, $Å^{-3}$	1.388 and -1.132

Table S10. Atomic coordinates, Wyckoff positions, and site occupancies in the crystal structure of $Cs_2InCl_5{\cdot}H_2O$ single crystal.

Atom	Position	*x/a*	*y/b*	*z/c*	Occ.
Cs	8*d*	0.35723(3)	-0.00191(3)	0.33844(6)	1
In	4*c*	0.11554(4)	1/4	0.30963(8)	1
Cl1	4*c*	0.24829(14)	1/4	0.0954(3)	1
Cl2	4*c*	0.00541(14)	1/4	0.0520(3)	1
Cl3	8*d*	0.10198(12)	0.00944(13)	0.3280(2)	1
Cl4	4*c*	0.22416(16)	1/4	0.5664(3)	1
O	4*c*	-0.0064(4)	1/4	0.5102(9)	1

Table S11. Anisotropic displacement parameters obtained from single-crystal structure refinement of $Cs_2InCl_5{\cdot}H_2O$

Atom	$U_{eq.}$, Å^2	U_{11}	U_{22}	U_{33}	U_{23}	U_{13}	U_{12}
Cs	0.03316(15)	0.0332(2)	0.0306(2)	0.0357(2)	0.00267(13)	-0.00306(14)	-0.00137(14)
In	0.02421(16)	0.0251(3)	0.0208(2)	0.0268(3)	0	0.0020(2)	0
Cl1	0.0281(4)	0.0272(9)	0.0298(9)	0.0274(9)	0	0.0044(8)	0
Cl2	0.0340(4)	0.0311(10)	0.0297(9)	0.0411(10)	0	-0.0070(9)	0
Cl3	0.0330(3)	0.0366(8)	0.0216(6)	0.0409(9)	0.0020(5)	0.0033(6)	-0.0015(5)
Cl4	0.0349(4)	0.0422(12)	0.0341(10)	0.0284(9)	0	-0.0042(8)	0
O	0.0451(18)	0.040(4)	0.035(3)	0.060(5)	0	0.027(4)	0

$U_{eq.}$ is defined as one third of the trace of the orthogonalized U_{ij} tensor. The anisotropic temperature factor exponent takes the form: $-2\pi^2[h^2a^{*2}U_{11} + ... + 2hka^*b^*U_{12}]$

Table S12. Interatomic distances (d, Å) and coordination numbers (C.N.) of the atoms of the $Cs_2InCl_5{\cdot}H_2O$

Atoms		d, Å	C.N.
Cs	Cl1	3.5384(14)	10
	Cl1	3.5421(14)	
	Cl2	3.6098(16)	
	Cl2	3.4694(15)	
	Cl3	3.6715(16)	
	Cl3	3.6792(19)	
	Cl3	3.7361(19)	
	Cl3	3.8235(16)	
	Cl4	3.4708(15)	
	Cl4	3.6529(16)	
In	– Cl1	2.484(2)	5
	– Cl2	2.480(2)	
	– Cl3	2.5060(13)	
	– Cl4	2.462(2)	
	– O	2.299(6)	

Table S13. Results of crystal structure refinement of the $Cs_2InBr_5 \cdot H_2O$ powder

Empirical Formula	$Cs_2InBr_5 \cdot H_2O$
Sp. Gr	$Pnma$ (№62)
Unit cell parameters, Å	a = 14.9648(6), b = 10.9166(3), c = 7.7063(3)
V, Å^3	1258.9(2)
Number of atoms in cell	36
Calculated density (g/cm^3)	4.2003(5)
Absorption coefficient (1/cm)	804.05
Radiation and wavelenght (Å)	CuKα; 1.54185
Diffractometer	SmartLab
Mode of refinement	Full profile
Number of free parameters	24
R_I; R_p	0.1147; 0.3823
Scale factor	1.14(1)
Texture axis and parameter	[0 2 1] 2.82(5)

Table S14. Refined coordinates of atoms and their isotropic thermal parameters of the $Cs_2InBr_5 \cdot H_2O$ powder

Atom	Position	*x/a*	*y/b*	*z/c*	Occ.	$B_{isot.}$, Å^2
Cs	8*d*	0.3595(4)	0.9983(9)	0.8280(9)	1	1.96(14)
In	4*c*	0.6175(7)	1/4	0.6969(15)	1	1.4(3)
Br1	4*c*	0.2242(12)	1/4	0.062(3)	1	3.2(6)
Br2	4*c*	0.2537(11)	1/4	0.590(2)	1	1.0(4)
Br3	8*d*	0.9991(13)	1/4	0.543(3)	1	1.9(5)
Br4	4*c*	0.1021(6)	0.005(2)	0.817(2)	1	2.3(3)
O	4*c*	0.979(7)	1/4	0.989(13)	1	3.0(27)

Table S15. Interatomic distances and coordination numbers (C.N.) of atoms in the structures of the $Cs_2InBr_5 \cdot H_2O$ powder

Atoms		d, Å	C.N.
Cs	– Br1	3.830(15)	10
	– Br1	3.6403(15)	
	– Br2	3.685(13)	
	– Br2	3.751(14)	
	– Br3	3.81(2)	
	– Br3	3.595(15)	
	– Br4	3.802(11)	
	– Br4	3.847(11)	
	– Br4	3.799(15)	
	– Br4	3.987(15)	
In	– Br1	2.53(2)	6
	– Br2	2.60(2)	
	– Br3	2.68(2)	
	– 2Br4	2.682(14)	
	– O	2.59(9)	

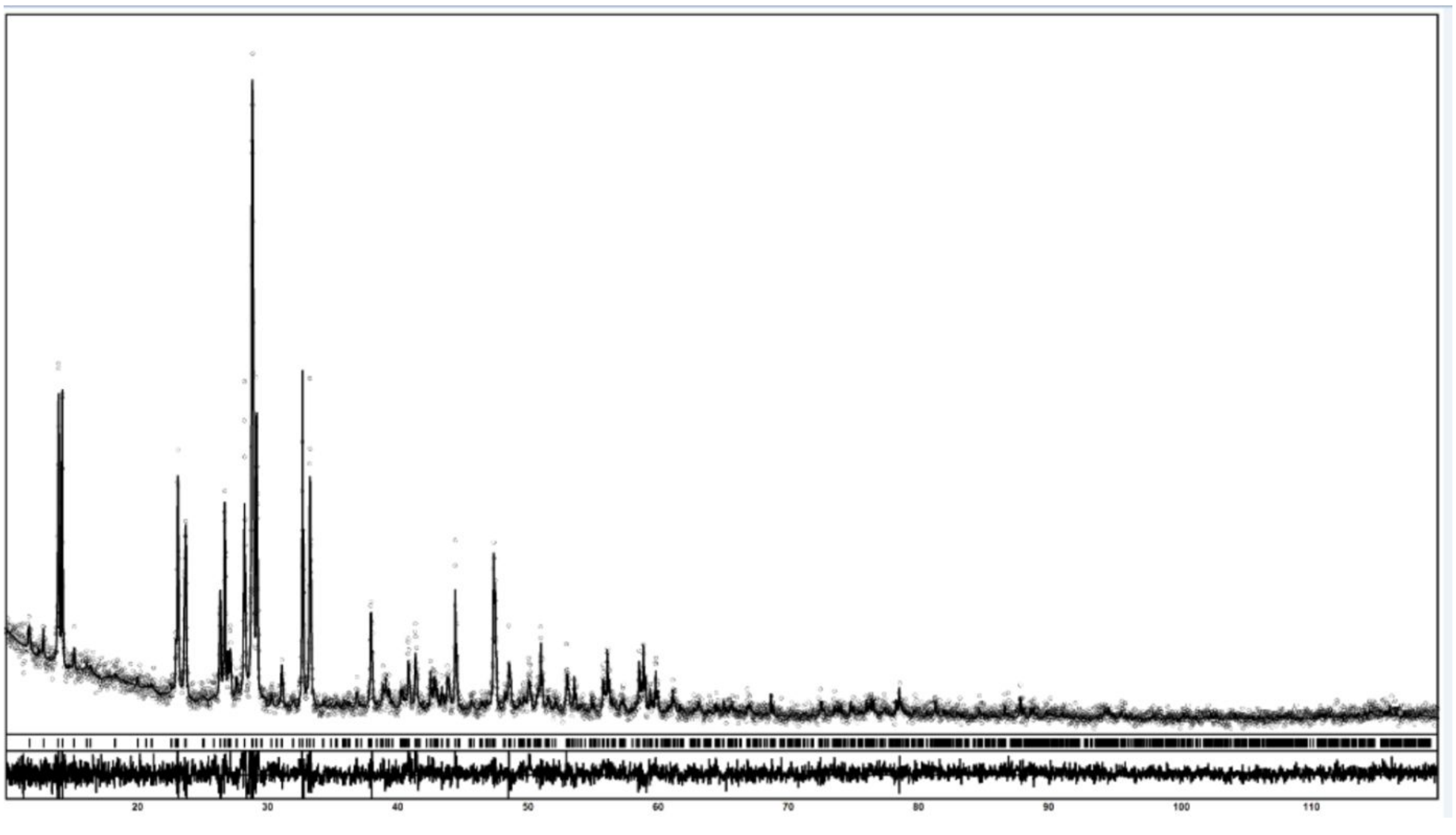

Fig. S8 Experimental (dots), calculated (solid) and difference (bottom scale) diffractograms of the $Cs_2InBr_5 \cdot H_2O$ powder sample.

Table S16. Binding energy values of core-level electrons (uncertainty is ±0.08 eV) as evaluated from the present XPS measurements made for the $Cs_2AgIn_{0.9}Sb_{0.1}Cl_6$ sample

Core-level	Binding energy (in eV)
In 4*d*	18.63
Cl 2*p*	198.7*
Ag $3d_{5/2}$	367.89
Ag $3d_{3/2}$	373.91
In $3d_{5/2}$	445.30
In $3d_{3/2}$	452.87
Cs $3d_{5/2}$	724.08
Cs $3d_{3/2}$	738.07

*Uncertainty is ±0.1 eV

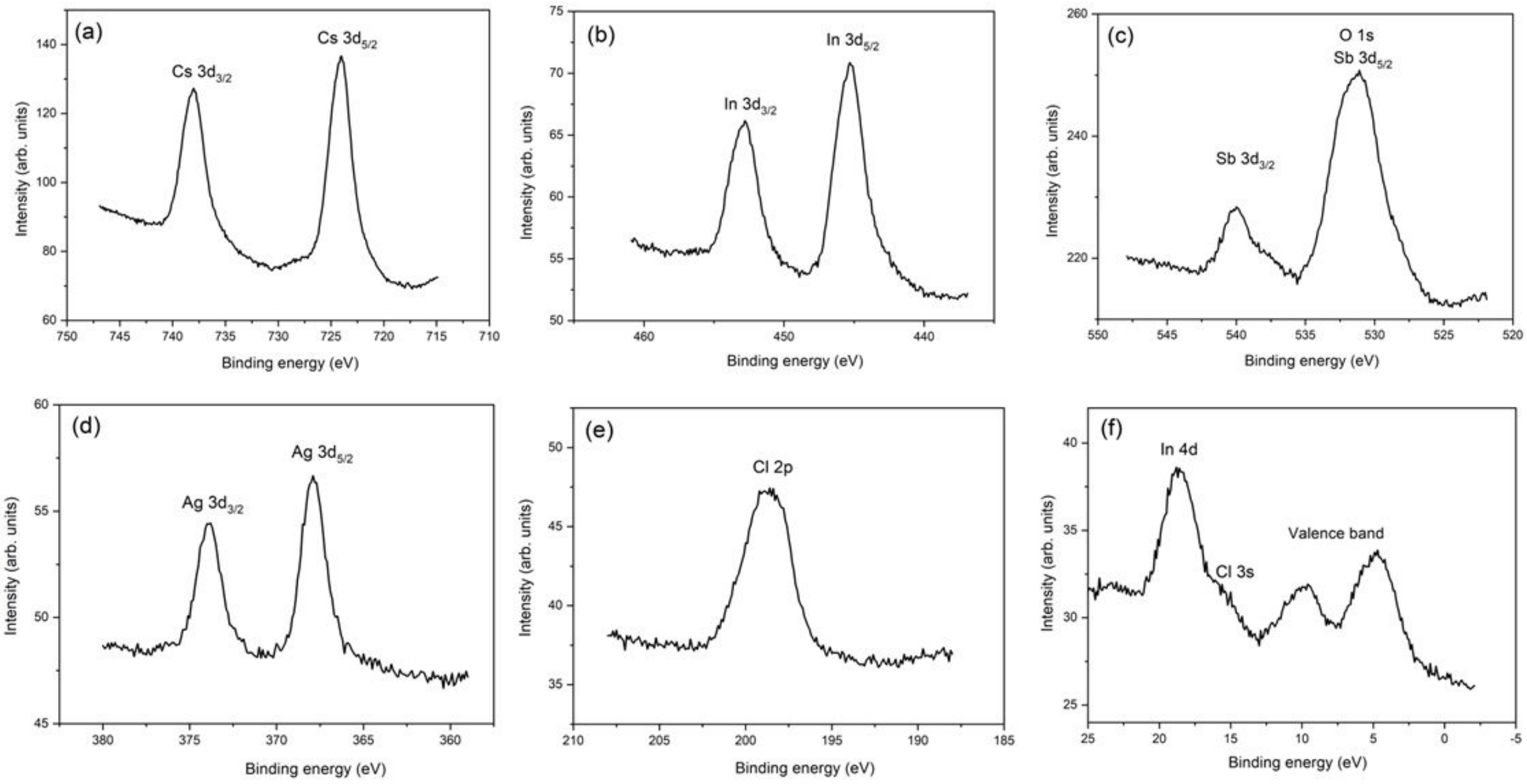

Fig. S9 The XPS core-level spectra of $Cs_2AgIn_{0.9}Sb_{0.1}Cl_6$: (a) Cs 3*d*, (b) In 3*d*, (c) O 1*s* + Sb 3*d*, (d) Ag 3*d*, (e) Cl 2*p*, and (f) valence-band region.

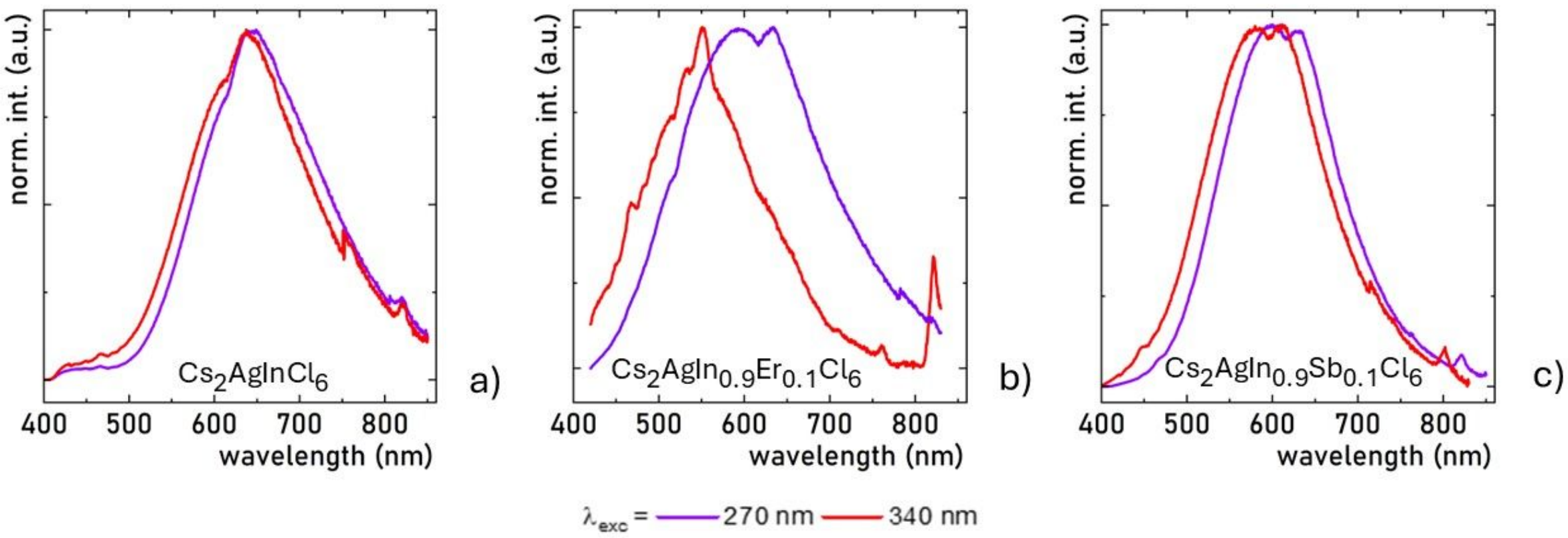

Fig. S10 The emission spectra obtained at λ_{exc} = 270 nm and 340 nm for: a) $Cs_2AgInCl_6$; (b) Er^{3+} doped; (c) Sb^{3+} doped samples.

References:

[1] P.W.M. Jacobs, Alkali halide crystals containing impurity ions with the n*s*^{2} ground-state electronic configuration, J. Phys. Chem. Solid. 52 (1991) 35–67

[2] L. Akselrud, Yu. Grin. WinCSD: software package for crystallographic calculations (Version 4) J. Appl. Crystallogr., 2014, 47, 803.

[3] G. M. Sheldrick Crystal structure refinement with SHELXL. Acta Cryst. C, 71, 2015, 3-8; https://shelx.uni-goettingen.de/

[4] G. Kresse, D. Joubert, From Ultrasoft Pseudopotentials to the Projector Augmented-Wave Method., Phys. Rev. B: Condens. Matter Mater. Phys. 1999, 59, 1758−1775.

[5] J. P. Perdew, K. Burke, M. Ernzerhof, Generalized Gradient Approximation Made Simple. Phys. Rev. Lett. 1996, 77, 3865−3868.

[6] H. J. Monkhorst, J. D. Pack, Special points for Brillouin-zone integrations, Phys. Rev. B. 13, 1976, 5188–5192.
